# Risk of emergency hospital admission related to adverse events after antibiotic treatment in adults with a common infection: impact of COVID-19 and derivation and validation of risk prediction models

**DOI:** 10.1186/s12916-024-03480-2

**Published:** 2024-07-02

**Authors:** Xiaomin Zhong, Victoria Palin, Darren M. Ashcroft, Ben Goldacre, Brian MacKenna, Amir Mehrkar, Sebastian C. J. Bacon, Jon Massey, Peter Inglesby, the OpenSAFELY collaborative, Kieran Hand, Alexander Pate, Tjeerd Pieter van Staa

**Affiliations:** 1 Centre for Health Informatics, School of Health Sciences, Faculty of Biology, Medicine, and Health, the University of Manchester, Manchester, M13 9PL UK; 2 Applied Health Research Unit, Nuffield Department of Population Health, Big Data Institute, Li Ka Shing Centre for Health Information and Discovery, University of Oxford, Oxford, OX3 7LF UK; 3 Maternal and Fetal Research Centre, Division of Developmental Biology and Medicine, the University of Manchester, St Marys Hospital, Oxford Road, Manchester, M13 9WL UK; 4 Centre for Pharmacoepidemiology and Drug Safety, School of Health Sciences, Faculty of Biology, Medicine and Health, University of Manchester, Oxford Road, Manchester, M13 9PL UK; 5 NIHR Greater Manchester Patient Safety Translational Research Centre, School of Health Sciences, Faculty of Biology, Medicine and Health, University of Manchester, Oxford Road, Manchester, M13 9PL UK; 6 Bennett Institute for Applied Data Science, Nuffield Department of Primary Care Health Sciences, University of Oxford, Oxford, OX2 6GG UK; 7 Pharmacy Department, Portsmouth Hospitals University NHS Trust, Portsmouth, UK; 8 NHS England, Wellington House, Waterloo Road, London, SE1 8UG UK

**Keywords:** Antibiotics, Adverse event, Common infection, COVID-19 pandemic

## Abstract

**Background:**

With the global challenge of antimicrobial resistance intensified during the COVID-19 pandemic, evaluating adverse events (AEs) post-antibiotic treatment for common infections is crucial. This study aims to examines the changes in incidence rates of AEs during the COVID-19 pandemic and predict AE risk following antibiotic prescriptions for common infections, considering their previous antibiotic exposure and other long-term clinical conditions.

**Methods:**

With the approval of NHS England, we used OpenSAFELY platform and analysed electronic health records from patients aged 18–110, prescribed antibiotics for urinary tract infection (UTI), lower respiratory tract infections (LRTI), upper respiratory tract infections (URTI), sinusitis, otitis externa, and otitis media between January 2019 and June 2023. We evaluated the temporal trends in the incidence rate of AEs for each infection, analysing monthly changes over time. The survival probability of emergency AE hospitalisation was estimated in each COVID-19 period (period 1: 1 January 2019 to 25 March 2020, period 2: 26 March 2020 to 8 March 2021, period 3: 9 March 2021 to 30 June 2023) using the Kaplan–Meier approach. Prognostic models, using Cox proportional hazards regression, were developed and validated to predict AE risk within 30 days post-prescription using the records in Period 1.

**Results:**

Out of 9.4 million patients who received antibiotics, 0.6% of UTI, 0.3% of URTI, and 0.5% of LRTI patients experienced AEs. UTI and LRTI patients demonstrated a higher risk of AEs, with a noted increase in AE incidence during the COVID-19 pandemic. Higher comorbidity and recent antibiotic use emerged as significant AE predictors. The developed models exhibited good calibration and discrimination, especially for UTIs and LRTIs, with a C-statistic above 0.70.

**Conclusions:**

The study reveals a variable incidence of AEs post-antibiotic treatment for common infections, with UTI and LRTI patients facing higher risks. AE risks varied between infections and COVID-19 periods. These findings underscore the necessity for cautious antibiotic prescribing and call for further exploration into the intricate dynamics between antibiotic use, AEs, and the pandemic.

**Supplementary Information:**

The online version contains supplementary material available at 10.1186/s12916-024-03480-2.

## Background

Antimicrobial stewardship is a long-term campaign to address the inappropriate use of antibiotics [1, 2]. In the UK, more than 80% of antibiotics are prescribed in primary care [3]. However, the prescription strategies vary across different practices, which also leads to concerns about inappropriate usage [4].

The unwarranted consumption of antibiotics is especially alarming due to its potential association with various adverse events, such as allergic responses, end-organ toxicity [5–8]. The identification of other harmful or adverse effects from antibiotics is becoming increasingly common [8]. Adverse effects of antibiotics can vary widely in frequency and severity, and may depend on the dosage or length of treatment, or they could be completely unpredictable. Often, neither the patient nor the prescriber recognises these direct harms, as the symptoms of the underlying illness or infection (such as nausea and vomiting) can obscure common side effects, which may go unreported by patients [9]. Therefore, overcoming those challenges and evaluating the risk of adverse events after antibiotic treatment becomes more essential.

Additionally, repeated exposure to antibiotics has been associated with increased risks of infection-related complications and more severe outcomes after a COVID-19 infection [10]. This underscores the importance of factoring in a patient’s previous antibiotic history when making clinical decisions [10, 11]. Studies highlight that implementing a more personalised evidence-based decision-making approach for antibiotic prescriptions could enhance patient care. The concept of prescribing based on a patient’s objective risk assessment and prognosis is gaining traction [12, 13].

The COVID-19 pandemic impacted primary care services, a recent study showed 66% of all adult consultation were remote in primary care during COVID-19, with remote consultations seeing a 1.23-fold increase in antibiotic prescriptions compared to face-to-face visits [14]. A recent study revealed that the alterations in antibiotic prescribing practices differed for various common infections and at distinct phases of the COVID-19 pandemic, and consultation rates for all common infections decreased [15]. However, within these reduced consultations, antibiotic prescribing patterns varied: prescriptions for lower respiratory tract infection (LRTI) decreased, those for upper respiratory tract infection (URTI) increased, while urinary tract infection (UTI) prescriptions remained stable. Additionally, except for UTI, there was an increase in the percentage of broad-spectrum antibiotics prescribed within these consultations [15–17]. Existing prognostic models in primary care predominantly concentrate on general adverse drug reactions and tend to prioritise elderly patients [18, 19]. There are no prediction tools specifically designed to assess the adverse effects of antibiotics for common infections at the primary care level, which also consider prior prescription history and individual patient comorbidities. This study addresses the gap in our current understanding of the impact of the COVID-19 pandemic on the incidence of adverse event (AEs) following antibiotic prescriptions for common infections and aims to predict the risk of developing AEs in this unique context.

The objective of this study was twofold: (1) to assess the impact of the COVID-19 pandemic on the incidence rates of AEs following antibiotic prescriptions for common infections and (2) to develop and validate predictive models for AEs in the context of the pandemic, considering patients’ long-term comorbidities and their history of short-term and long-term antibiotic use. The study predominantly focuses on six common infections: UTI, LRTI, URTI, sinusitis, otitis externa, and otitis media.

## Methods

All data were linked, stored, and analysed securely using the OpenSAFELY platform, https://www.opensafely.org/, as part of the NHS England OpenSAFELY COVID-19 service. Data included pseudonymised data such as coded diagnoses, medications, and physiological parameters. No free text data are included. All code is shared openly for review and re-use under MIT open license (https://github.com/opensafely/amr-uom-brit). Detailed pseudonymised patient data is potentially re-identifiable and therefore not shared.

Primary care records managed by the GP software provider TPP, which provides almost 24 million peoples electronic health records (EHRs), were linked to hospital admission data from the NHS Digital Secondary Use Service (SUS), through OpenSAFELY. Information about COVID-19 test results were linked to two sources: the Second Generation Surveillance System (SGSS) and the primary care records of COVID-19 diagnosis. SNOMED CT codes were employed to extract the records with common infections.

### Study population

The population for our study encompassed all adults aged 18 to 110 years with recorded sex and region and who were registered as active patients in a TPP practice from January 2019 to June 2023. The study duration was segmented based on the implementation of national lockdowns: (1) period 1 from 1 January 2019 to 25 March 2020, (2) period 2 from 26 March 2020 to 8 March 2021, and (3) period 3 from 9 March 2021 to 30 June 2023. To guarantee that baseline characteristics were accurately recorded, patients with less than 3 months of prior follow-up at the onset of each designated period were excluded.

We extracted the antibiotic user cohort, comprising patients with at least one antibiotic prescription during the study period. The recorded date of the antibiotic prescription was designated as the index date. Since this study aimed to predict the risk of adverse events after taking antibiotics for common infections (UTI, LRTI, URTI including coughs, colds, and sore throats, sinusitis, otitis externa, and otitis media), we excluded patients without any code in the records for a common infection at the date of the antibiotic prescription or in the 30 days before. Patients with chronic respiratory disease history were excluded due to their frequent use of antibiotic rescue packs, which muddles the association between the timing of use and the prescription date. To minimise any impact of a COVID-19 infection on hospitalisation, any patient with a positive SARS-CoV-2 test ± 6 weeks from the infection record date was excluded.

### Outcomes

The outcome measured was an emergency hospitalisation with an admission code denoting the reason for admission [20] for AEs, which could potentially signal adverse drug reactions (ADRs) or side-effects to antibiotics. Patients were followed for 30 days after the index date. In case of a repeat antibiotic prescription within these 30 days, follow-up for the initial prescription ended at the date of repeat prescription and follow-up for the subsequent prescription was reset to 30 days. We employed a codelist derived from a systematic search and evaluation of lists in 41 publications that identified ADRs from administrative data [21]. This review categorised codes based on the likely causality level as indicated by the ICD-10 code including (i) ICD-10 codes with the phrase ‘induced by medication/drug,’ (ii) ICD-10 codes with the phrase ‘induced by medication or other causes’ or ‘poisoning by medication,’ (iii) ADRs considered to be very likely, or (iv) likely, even though the ICD-10 code description does not reference a drug [21]. In our study, codes referring to a drug other than an antibiotic or with an evident non-antibiotic related reason were omitted (e.g. F11 mental and behavioural disorders due to opioid use). Our study focused on incident events; patients with the same outcome 1 year before were excluded.

### Predictor variables

The full list of potential predictors was compiled based on previous studies and consultations with clinical experts; this list is detailed in Additional file 1: Tab. S1. We extracted patient-level characteristic variables, including age, sex, ethnicity (white, mixed, south Asian, black, other), smoking status (current, former, never), and the Index of Multiple Deprivation (IMD) quintile. BMI was categorised into six groups (plus one group for missing data) according to the NICE definition: not obese (< 30 kg/m^2^), obese I (30–34.9 kg/m^2^), obese II (35–39.9 kg/m^2^), and obese III (≥ 40 kg/m^2^) [22]. Health status variables were assessed in the most recent 5 years (prior to the index date) and categorised according to the Charlson Comorbidity Index (CCI): no comorbidities, low, medium, high, and very high [23]. The antibiotics included in this study were based on the British National Formulary (BNF) chapter 5.1 (Antibacterial Drugs). Antituberculosis drugs (BNF 5.1.9) and antileprotic drugs (BNF 5.1.10) were excluded from the study. The code list for antibiotics is available in Table S1. Antibiotic history was represented by two predictors: one was the number of antibiotic prescriptions in the last 3 years (3 years plus 90 days to 90 days before the index date): 0, 1, 2–3, and 4 + , and a binary variable indicating recent antibiotic prescription 30 days before the index date.

### Statistical methods

The cohorts for common infections were divided by infection type. To evaluate the trends impact by COVID-19 pandemic, the study calculated the incidence rate of AEs by each infection and monthly trends over time. Additionally, the survival probability group by different time period (before, during, and after the pandemic) of emergency AE hospitalisation was estimated in each split sub-dataset using the Kaplan–Meier approach. This estimation process was conducted across various time periods. Cox proportional hazards regression models were applied to both the pre-pandemic and overall cohorts. Patients entered the study after receiving an antibiotic prescription from a GP for one of the common infections and were monitored for the subsequent 30 days. Censored patients were those who died or were lost to follow-up (whichever came first). In the case of patients with multiple antibiotic prescriptions, each prescription was included into the analysis. In the case of a repeat antibiotic prescription within 30 days, follow-up of the first prescription was stopped at the date of subsequent prescription. Patients with missing values for ethnicity, smoking status, IMD, and BMI variables were assigned a missing indicator labelled ‘Unknown’. The models were adjusted with missing indicators to increase the accuracy and reduce bias [24]. Each sub-dataset for each common infection was randomly divided into development (75%) and validation (25%) cohorts and used to develop and validate a set of Cox models for common infections (in instances where a single patient has multiple prescriptions, they will be allocated to either the development or the validation cohort, but not both). Age was modelled using a restricted cubic spline, and estimated log hazard ratios (HRs) against continuous age were plotted. Additionally, HRs for specified age brackets (40–49, 50–59, 60–69, 70–79, and 80 + years, each compared with the 18–39 years reference group) were computed from models where age was integrated as a categorical variable, rather than through a cubic spline. This modelling process was reiterated for both the sub-cohort within period 1 and the entirety of the cohort, separately. Notably, the patient profiles constituting the development dataset in period 1 mirrored those in the development dataset of the overall cohort. To investigate the impact of the COVID-19 pandemic, we included a categorical variable representing the different time period.

The performance of the models was evaluated in terms of discrimination and calibration, as recommended by the Practical Guidance for Cox Proportional Hazards Models and the TRIPOD (Transparent Reporting of a Multivariable Prediction Model for Individual Prognosis or Diagnosis, Additional file 2) statement [25, 26]. The ability to discriminate was assessed using the concordance statistic (c-statistic) in both the development and validation datasets. Calibration was evaluated by plotting the observed risk of AE emergency admission against the predicted risk grouped by deciles of the predicted risks [27]. The resulting curve was compared to a model with ideal calibration, which is characterised by a calibration in-the-large (intercept) of 0 and a calibration slope of 1.

## Results

**Fig. 1:**
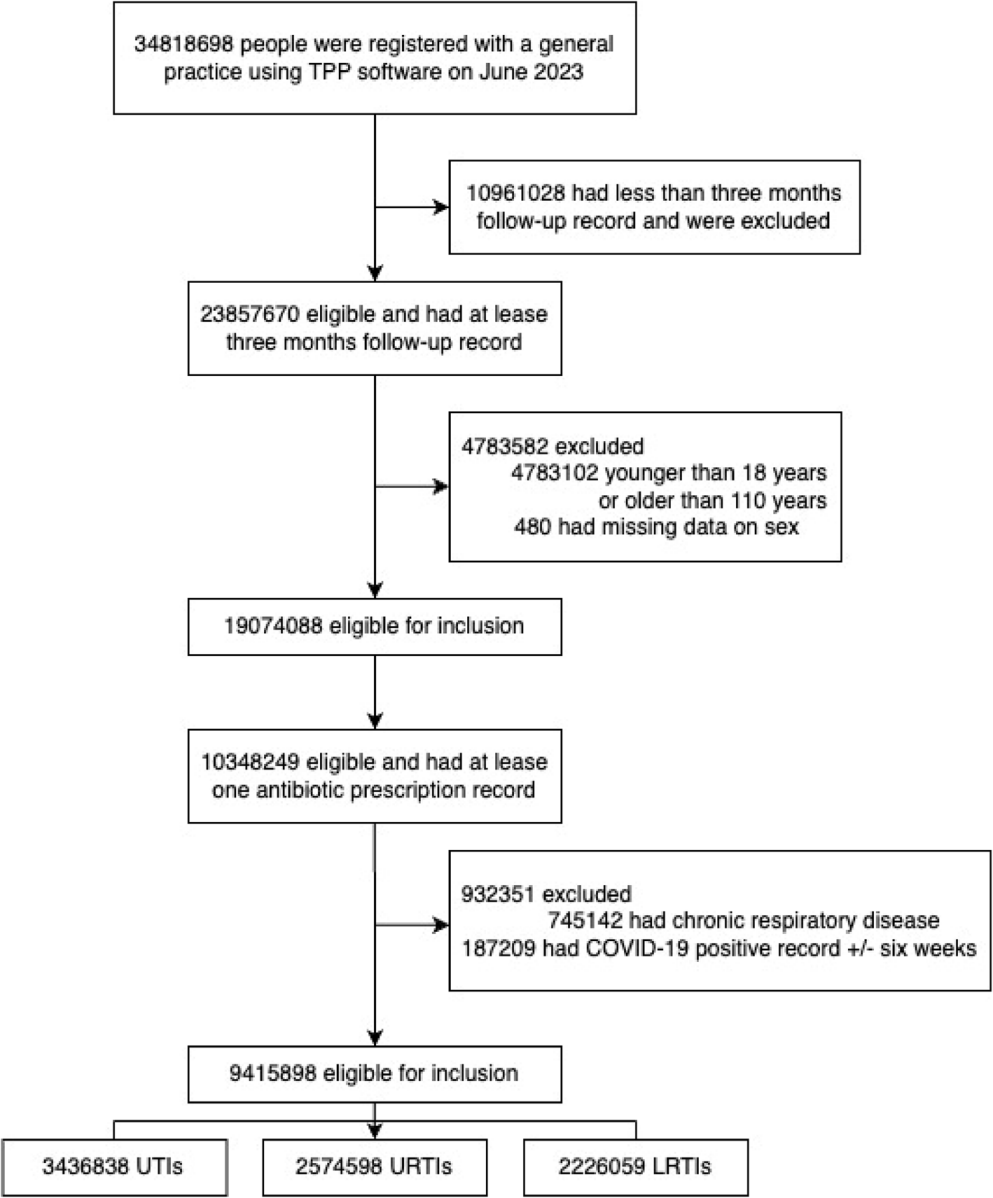
The flowchart of participant selection

Throughout the study period, a total of 9,415,898 eligible patients received prescriptions (Fig. 1). Of these, 46.4% had a recorded consultation for an infection in the 30 days preceding their prescription (including the same-day infection record). A breakdown of these prescriptions indicates that 3,436,838 patients had a UTI record, while 2,574,598 were noted for URTI, and 2,226,059 for LRTI. When examining the incidence of adverse events post-antibiotic treatment, variations were observed across different infections. Specifically, 0.6% (19,914 patients) with UTI, 0.3% (7187 patients) with URTI, and 0.5% (10,536 patients) with LRTI experienced such events (Table 1, see Additional file 1: Tab. S2 for otitis externa, otitis media, and sinusitis). It was found that 37.2% of UTI patients had another antibiotic prescription record within 30 days before this identified prescription, 22.8% for URTIs and 29.3% for LRTIs. In UTI patients, the most common adverse effects were kidney problems (21.3%), with 0.7% related to the liver. Another 0.9% were recorded as poisoning. In URTI and LRTI, these involved the circulatory system (22.7% and 27.5%, respectively), with acute kidney diseases accounting for 8.3% in URTI and 12.3% in LRTI. Acute liver issues comprised 0.4% in URTI and 0.5% in LRTI (see Additional file 1: Tab. S3).

**Table 1:** Patient characteristics. Summary statistics are number (percentage) except where indicated

Characteristic	Levels	UTI	URTI	LRTI
Total *N*		3436838	2574598	2226059
Event	Yes	19914 (0.6)	7187 (0.3)	10536 (0.5)
	No	3416924 (99.4)	2567411 (99.7)	2215523 (99.5)
Age	Mean (SD)	58.5 (21.1)	47.4 (19.8)	57.8 (19.3)
	18–39	818810 (23.8)	1081326 (42.0)	462981 (20.8)
	40–49	367856 (10.7)	369393 (14.3)	298800 (13.4)
	50–59	473793 (13.8)	376199 (14.6)	394408 (17.7)
	60–69	498051 (14.5)	318445 (12.4)	387831 (17.4)
	70–79	636205 (18.5)	249238 (9.7)	354507 (15.9)
	80 +	642123 (18.7)	179997 (7.0)	327532 (14.7)
Sex	Women	2842140 (82.7)	1713699 (66.6)	1383325 (62.1)
	Men	594698 (17.3)	860899 (33.4)	842734 (37.9)
IMD^1^	5 (least deprived)	692209 (20.1)	426804 (16.6)	378476 (17.0)
	4	724293 (21.1)	481774 (18.7)	433210 (19.5)
	3	739066 (21.5)	535850 (20.8)	463903 (20.8)
	2	645309 (18.8)	542790 (21.1)	448278 (20.1)
	1 (most deprived)	629954 (18.3)	583028 (22.6)	499090 (22.4)
	Unknown	6007 (0.2)	4352 (0.2)	3102 (0.1)
Ethnicity^2^	White	3077914 (89.6)	2137161 (83.0)	1962577 (88.2)
	Mixed	27732 (0.8)	31230 (1.2)	16955 (0.8)
	South Asian	176148 (5.1)	231926 (9.0)	140744 (6.3)
	Black	41596 (1.2)	47869 (1.9)	27461 (1.2)
	Other	37018 (1.1)	38777 (1.5)	22274 (1.0)
	Unknown	76430 (2.2)	87635 (3.4)	56048 (2.5)
Region	East of England	817835 (23.8)	657957 (25.6)	525098 (23.6)
	North East	142631 (4.2)	116764 (4.5)	110589 (5.0)
	North West	361768 (10.5)	245323 (9.5)	244854 (11.0)
	Yorkshire and the Humber	527111 (15.3)	398315 (15.5)	383917 (17.2)
	East Midlands	672547 (19.6)	457378 (17.8)	433304 (19.5)
	West Midlands	143416 (4.2)	132497 (5.1)	101774 (4.6)
	London	120411 (3.5)	157510 (6.1)	68005 (3.1)
	South East	221040 (6.4)	150588 (5.8)	129481 (5.8)
	South West	427004 (12.4)	256106 (9.9)	228059 (10.2)
	Unknown	3075 (0.1)	2160 (0.1)	978 (0.0)
Smoking^3^	Never and unknown	1615490 (47.0)	1,203718 (46.8)	875143 (39.3)
	Former	1429,954 (41.6)	906490 (35.2)	931720 (41.9)
	Current	391394 (11.4)	464390 (18.0)	419196 (18.8)
BMI^4^	Not obese	2480753 (72.2)	1726133 (67.0)	1434774 (64.5)
	Obese I (30–34.9 kg/m^2^)	541513 (15.8)	441434 (17.1)	417910 (18.8)
	Obese II (35–39.9 kg/m^2^)	243425 (7.1)	228806 (8.8)	210241 (9.4)
	Obese III (40 + kg/m^2^)	171147 (5.0)	180225 (7.0)	163134 (7.3)
CCI^5^	No	2073726 (60.3)	1697263 (65.9)	1198348 (53.8)
	Low	1197540 (34.8)	804806 (31.3)	914397 (41.1)
	Medium	151911 (4.4)	67468 (2.6)	104656 (4.7)
	High	13103 (0.4)	4886 (0.2)	8308 (0.4)
	Very high	558 (0.0)	175 (0.0)	350 (0.0)
Antibiotic history (3 years)^6^	0	586477 (17.1)	670900 (26.1)	527497 (23.7)
	1	457313 (13.3)	463755 (18.0)	372012 (16.7)
	2–3	695916 (20.2)	590510 (22.9)	496792 (22.3)
	4 +	1697132 (49.4)	849433 (33.0)	829758 (37.3)
Antibiotic use (30 days)^7^	Yes	1279252 (37.2)	586501 (22.8)	652392 (29.3)
	No	2157586 (62.8)	1988097 (77.2)	1573667 (70.7)

^1^IMD (Index of Multiple Deprivation) quintile measured from patient-level address

^2^Ethnicity in line with 2001 Census categories

^3^Smoking status identified from the most recent clinical records

^4^BMI, body mass index groups based on the NICE definitions

^5^The Charlson Comorbidities Index (CCI) is a method of categorising comorbidities of patients based on the International Classification of Diseases (ICD) diagnosis codes found in administrative data. It includes 17 weighted conditions such as myocardial infarction, congestive heart failure, peripheral vascular disease, cerebrovascular disease, dementia, chronic pulmonary disease, connective tissue disease, ulcer disease, mild liver disease, diabetes, hemiplegia, moderate or severe renal disease, diabetes with complications, any malignancy (including leukaemia and lymphoma), moderate or severe liver disease, metastatic solid tumour, and AIDS

^6^The patient’s antibiotic prescription history spans from three years plus 90 days, up until 90 days prior to the outcome date

^7^The binary variable indicating if there were any antibiotic treatments administered in the 30 days preceding the index date

**Fig. 2:**
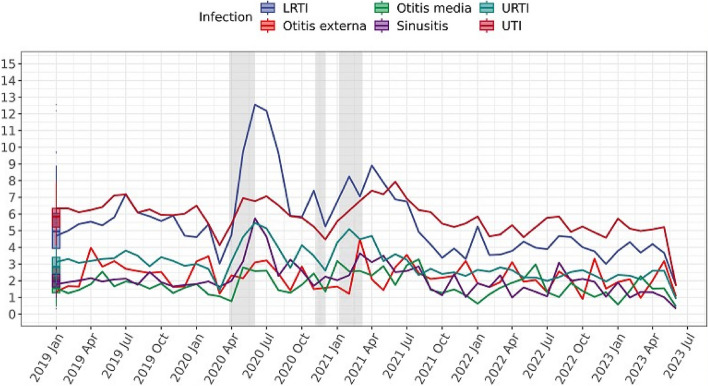
Incidence rates of AE over time calculated every month based on the number of new cases per 1000 patients at risk (antibiotic users with certain infection consultation). Numerator is the number of adverse event cases (times 1000), and the denominator is the number of patients at risk, grouped by infection type. Boxplots represent the historical average (median and IQR) percentage of incidence rates of new AE’s cases from January 2019 to June 2023. The shadow area indicating the periods of national lockdown

**Fig. 3:**
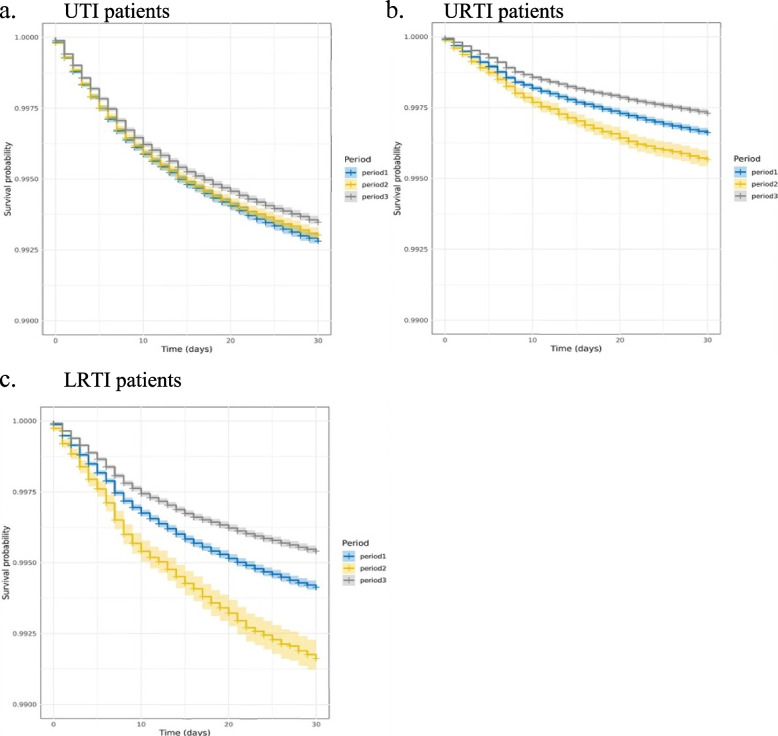
Kaplan–Meier plots for AE in 30 days after antibiotics. Plots show cumulative survival probability of AE by period and infection. The study duration was segmented based on the implementation of national lockdowns: (1) period 1 from 1 January 2019 to 25 March 2020, (2) period 2 from 26 March 2020 to 8 March 2021, and (3) period 3 from 9 March 2021 to 30 June 2023

Figure 2 shows the incidence rate of AEs by each infection and monthly trends over time. UTI and LRTI patients showed higher risks in developing AE. Apart from UTIs, which remained relatively stable over time, the incidence rates of AEs in all other infections observed an increase between April and June 2020. Additionally, there was a higher incidence in period 1 and period 3 for UTI patients, but LRTI patients had the highest risk in period 2. Kaplan–Meier curves showed that the occurrence of AEs is more pronounced within the initial 10 days following antibiotic administration compared to the subsequent 20 days (Fig. 3). Figure 3 also shows that there was an increased 30-day incidence during period 2, a trend consistently observed across most common infections, excluding UTIs and otitis externa (Additional file 1: Fig. S1).

**Fig. 4:**
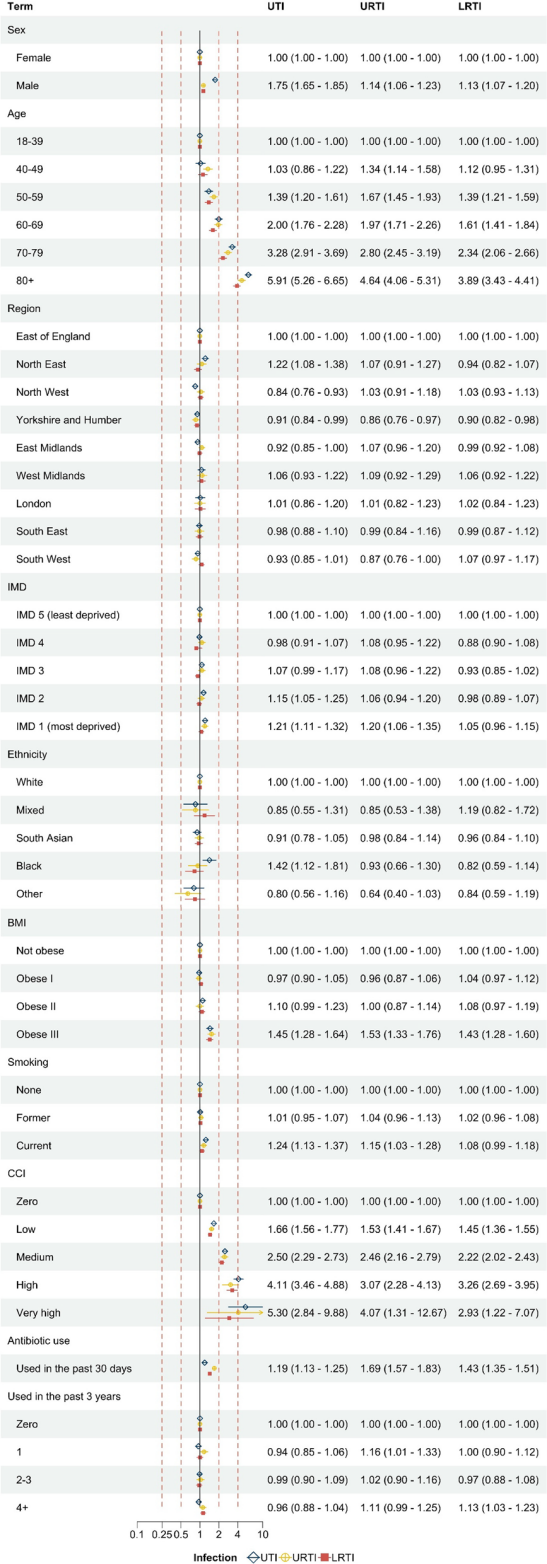
Period 1 cohort (pre-COVID): adjusted hazard ratios for selected predictors (including health behavioural and clinical variables). The Index of Multiple Deprivation (IMD) quintile was derived from the patient’s residential address. Body mass index (BMI) refers to a calculation of body fat based on height and weight. obese I (30–34.9 kg/m^2^), obese II (35–39.9 kg/m^2^), and obese III (≥ 40 kg/m^2^). The Charlson Comorbidities Index (CCI) is a method of categorising comorbidities of patients based on the International Classification of Diseases (ICD) diagnosis codes found in administrative data. It includes 17 weighted conditions such as myocardial infarction, congestive heart failure, peripheral vascular disease, cerebrovascular disease, dementia, chronic pulmonary disease, connective tissue disease, ulcer disease, mild liver disease, diabetes, hemiplegia, moderate or severe renal disease, diabetes with complications, any malignancy (including leukaemia and lymphoma), moderate or severe liver disease, metastatic solid tumour, and AIDS. Used in the past 30 days: the binary variable indicating if there was any antibiotic treatments administered in the 30 days preceding the index date. Used in the past 3 years: the patient’s antibiotic prescription history spans from three years plus 90 days, up until 90 days prior to the outcome date. Reference groups: Sex: Female, Age: 18–39, Region: East of England, IMD quintile: the least deprived quintile (IMD 5), Ethnicity: white, BMI: Not obese (< 30 kg/m^2^) Smoking: None (smoking status identified from the most recent clinical records), CCI: Zero, Antibiotic use: used in the past 30 days: No, used in the past 3 years: zero

Figure 4 reports the HRs of the predictors for each infection (models were trained and tested using the data from period 1, models for otitis externa, otitis media, sinusitis are reported in Additional file 1: Fig. S2). Due to varying incidence rates by COVID-19 (see Additional file 1: Fig. S3 and S4), we chose the models from period 1 as the final risk prediction model (baseline hazard and model coefficients are reported in Additional file 1: Tab. S4). The HRs for UTI, URTI, and LRTI are provided in Fig. 4. The other infections are shown in Additional file 1: Fig. S3. Age was observed as a key predictor, with relatively higher HRs in older age groups. The HRs were 5.91 (5.26–6.65) in UTI, 4.64 (4.06–5.31) in URTI, and 3.89 (3.43–4.41) in LRTI when comparing ages 80 + to 18–39. CCI and antibiotic usage in the past 30 days were also identified as important predictors. Specifically, the HRs were significant: for the ‘Very high’ CCI category compared to the ‘Zero’ category, the HRs were 5.30 (2.84–9.88) in UTI, 4.07 (1.31–12.67) in URTI, and 2.93 (1.22–7.07) in LRTI. Additionally, patients who received another antibiotic prescription within 30 days before the current prescription demonstrated higher HRs as well: 1.19 (1.13–1.25) for UTI, 1.69 (1.57–1.83) for URTI, and 1.43 (1.35–1.51) for LRTI, respectively.

**Fig. 5:**
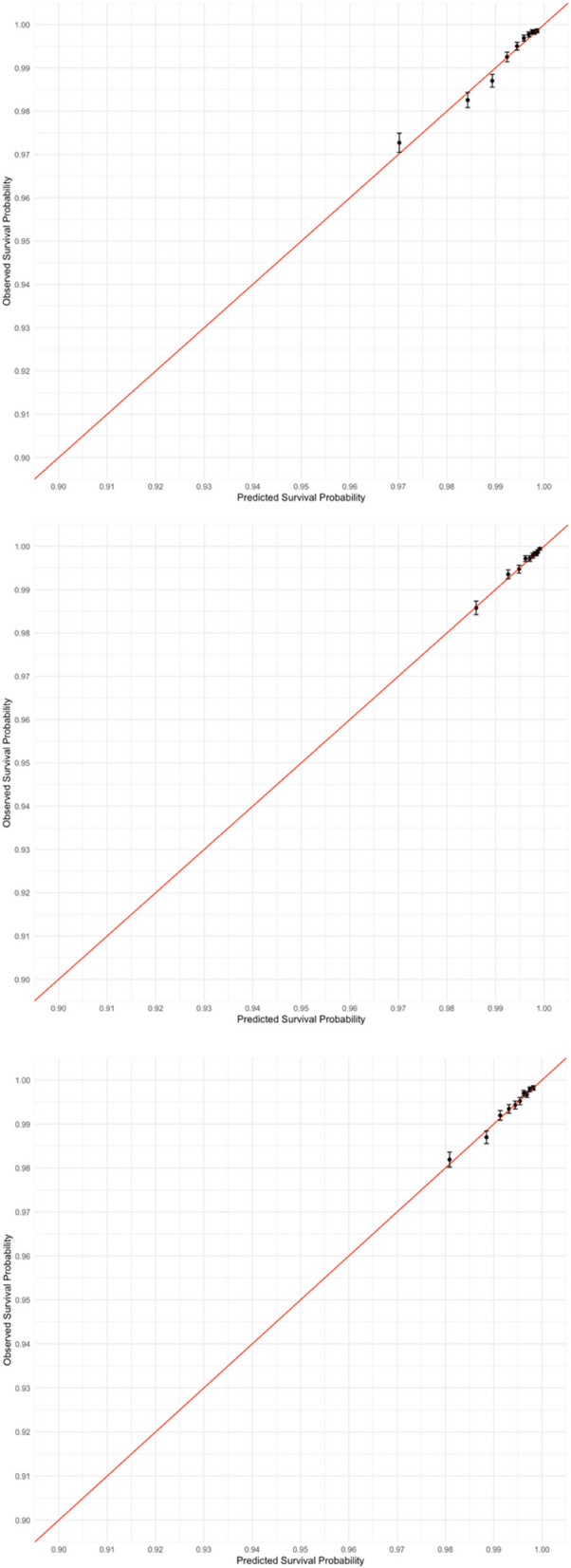
Calibration plot for UTI/URTI/LRTI models. Calibration plot showing observed survival probabilities (*Y*-axis) versus predicted survival probabilities (*X*-axis). The plot was generated from the validation cohort

The predictor age modelled with restricted cubic splines was reported in Additional file 1: Fig. S5, and we observed that older patient had a higher HRs for developing AE. The calibration among all models were good (Fig. 5 and Additional file 1: Fig. S6), with near perfect agreement between the predicted and observed risks across the entire range of predicted risk. This is supported by the calibration slope of 1.011 in UTI model (validation dataset) and 1.022 in URTI model and 0.983 in LRTI model, respectively (Table 2).

**Table 2:** Model performance at day 30: calibration and discrimination (pre-pandemic cohort, with confidence intervals calculated by bootstrap)

Infection		C-statistic	Calibration slope
UTI	Development	0.76 (0.76–0.77)	0.999
	Validation	0.76 (0.75–0.77)	1.011
URTI	Development	0.73 (0.72–0.74)	1.000
	Validation	0.73 (0.72–0.75)	1.022
LRTI	Development	0.70 (0.69–0.71)	1.000
	Validation	0.70 (0.68–0.71)	0.983
Otitis externa	Development	0.75 (0.72–0.78)	1.000
	Validation	0.72 (0.66–0.78)	0.920
Otitis media	Development	0.72 (0.68–0.76)	1.000
	Validation	0.70 (0.62–0.77)	0.864
Sinusitis	Development	0.65 (0.62–0.67)	1.000
	Validation	0.69 (0.65–0.74)	1.169

The model’s discrimination was evaluated using the C-statistic, as reported in Table 2. The performance of the model, which was trained and validated on the overall cohort, is detailed in Additional file 1: Tab. S5. In the validation cohort, 5 of 6 models exhibited good levels of C-statistics (> 0.70) in predicting AEs, except for sinusitis (0.69 (0.65–0.74), see Table 2). The UTI model had the highest C-statistic of 0.76 (0.75–0.77).

## Discussion

This study examined a substantial cohort of patients who were prescribed antibiotics following consultations for common infections. A varied incidence of AEs was observed across different types of infections, with particular prominence for more severe infections such as UTI and LRTI. Notably, temporal trends in AE incidence were found to differ depending on the infection type and specific periods defined by the COVID-19 pandemic, suggesting a possible impact between the pandemic and AE risk. Our models, particularly those based on UTI and LRTI patient data, demonstrated good discriminative power as assessed by C-statistic values. Key predictors, such as the CCI and recent antibiotic usage, emerged as significant factors contributing to AE risk. Calibration of these predictive models was robust, providing reliable estimates across a range of predicted risks.

Our study provides valuable insights into the incidence of AEs following antibiotic prescriptions for common infections. As a key predictor, elderly patients had higher risks of experiencing emergency admissions for adverse events. Along with other existing evidence that infections in elderly patients are more likely to progress and develop infection-related complications, better monitoring and more personalised strategies are recommended [11]. CCI and recent antibiotic usage emerged as significant predictors for AE risk, corroborating earlier studies that identified comorbidities and prior antibiotic exposure as risk factors [28]. A study by Aldeyab et al. demonstrated that there is a significant positive relationship between antibiotic use and the sum of age-adjusted comorbidity scores. This suggests that individuals with higher comorbidity scores, which often include various chronic conditions, are more likely to use antibiotics and subsequently may experience AEs [29]. Moreover, recent studies noticed that inappropriate antibiotic use has been associated with an increased risk of adverse reaction [30, 31]. The robustness of our predictive models, especially for UTI and LRTI patients, adds to the growing body of evidence supporting the use of predictive analytics in healthcare.

Interestingly, our study found that the COVID-19 pandemic had a variable impact on AE incidence rates depending on the type of infection. This suggests a complex interplay between the pandemic and antibiotic-related AEs, warranting further investigation. Explanations may be that during the pandemic the capacity in microbiological diagnosis reduced and that clinicians were more likely to prescribe broad-spectrum antibiotics. In April 2020, 40% of COVID-19 positive patients were given antibiotics, and about 20% of those antibiotics were broad-spectrum. However, this number reduced to 20% in June 2020 after the rapid guideline was published suggesting not to prescribe antibiotics to COVID-19 positive patients [32]. Existing studies found that the percentage of broad-spectrum antibiotics increased at the beginning of the pandemic and returned to pre-pandemic levels by the end of 2021 [33].

In this study, we predicted the risk of developing AEs by using data from both the pre-pandemic period and the overall cohort. This approach provides us with a better understanding of the specific risks associated with taking antibiotics for various common infections across different time periods. However, changes in healthcare delivery in primary care practices, such as the adoption of virtual consultations, increased prescribing rates, and prioritisation of certain patient groups, may have significantly altered the landscape [14–17]. Additionally, post-pandemic observations indicate that healthcare delivery has recovered to pre-pandemic levels in various aspects [16, 31, 34]. The models developed using pre-pandemic data are suggested for future use in predicting the risk of AE.

The findings may have several implications for clinical practice. First, healthcare providers should exercise caution when prescribing antibiotics, particularly for UTI and LRTI, and consider patient-specific factors like CCI and recent antibiotic usage [35]. Second, the variable impact of the COVID-19 pandemic on AE rates suggests that healthcare systems should be prepared for fluctuating AE incidence during public health crises [36]. Previous studies have identified an association between increased frequencies of past antibiotic exposure and a heightened risk of complications arising from infections and autoimmune diseases [37, 38]. One prevailing hypothesis suggests that routine antibiotic use may elevate the likelihood of patients becoming colonised and subsequently infected by antibiotic-resistant pathogens. This scenario may lead to the failure of antibiotic treatments and increased vulnerability to the harmful effects of infections [10, 39]. However, our study found that, in cases of otitis externa, otitis media, and sinusitis, long-term antibiotic exposure played a more substantial role in predicting AEs than in other infections. As headaches were commonly reported as a disease-related event among the three infections above, this might suggest that headaches may be more related to infection-related complications in patients with decreased antibiotic efficacy due to prior extensive use, rather than being a direct side effect of the antibiotics themselves. The study also found that in less severe infections like URTI, the harm-benefit ratio may not be as favourable. Although there are only slight reductions in the risk of severe infection-related complications, there is an increased risk of acute renal failure. Our study revealed that AEs might occur in various organs including the liver, kidneys, and other parts of the genitourinary system. About 10.2% to 11.0% of AEs were observed in the digestive system. These could not be clearly distinguished as direct harms or symptoms of the underlying illness or infection. However, another study we conducted showed that patients prescribed antibiotics other than first-line treatments, particularly broad-spectrum antibiotics, have a higher odds of developing AEs [40]. This provides evidence of the need to select antibiotics with a more favourable harm-benefit ratio. Frequent use of antibiotics can alter the microbiome, leading to increased resistance. This shift may necessitate the use of more potent and potentially more toxic antibiotics in subsequent infections, further increasing the risk of AEs [41].

Prior epidemiological studies have shown that a history of frequent antibiotic prescriptions is linked to elevated risks of complications related to infections [42]. Although confounding factors could account for these results, growing evidence suggests that antibiotics negatively impact microbiota, including those in the respiratory tract, thereby weakening the host microbiota’s defence against harmful microorganisms [43, 44]. Krockow et al. emphasised the need for effective strategies, such as behavioural interventions, to minimise repetitive antibiotic prescribing [45]. This underscores the significance of incorporating well-informed support tools in clinical decision-making [12]. In this study, developed prediction models will be incorporated into a Knowledge Support System (KSS) intervention, utilising a Learning Healthcare System (LHS) approach for antibiotic prescriptions related to common infections in primary care [12, 46, 47]. We conducted mixed-method co-design workshops with clinicians to assess the model’s acceptability among prescribing healthcare professionals and to identify key factors that could enhance uptake [48]. The feedback highlighted various important elements, such as extracting key information from care records (including the history of antibiotic prescriptions), suggested actions, personalised treatment plans, and risk indicators. These indicators include risks of patient emergency admission due to adverse events or infection-related complications, the risk of repeat prescriptions (potentially due to antibiotic failure), and the content suitable for patient information sheets [11, 31, 49].

While our study offers important contributions, it is not without limitations. The data are observational and thus cannot establish causality. Additionally, the study did not account for other potential confounding variables such as patient adherence to medication, which could influence AE incidence [50]. In spite of our efforts to account for existing illnesses by adjusting for comorbid conditions, assessing the severity of specific diseases using the EHRs at hand proves difficult. However, it is impractical to anticipate that randomised studies will be carried out to explore the broad range of adverse events examined in this research. As a result, the observational findings of this study should be viewed alongside evidence from broader sources and the likelihood of possible causal links. In this study, we aimed to enhance decision-making for clinicians presented with common infections by examining antibiotic prescriptions on the date of infection diagnosis. However, it is important to consider that this approach may not capture those patients whose infection diagnoses were recorded a few days post-antibiotic prescriptions, as noted in studies by Palms et al. and Olesen et al. [51, 52]. Nevertheless, our prior research involving the same population revealed no differences in the patterns between same-day prescriptions and those within a ± 7-day window around the infection diagnosis [53]. These findings underscore our decision to adhere to the same-day time frame in the current study. Additionally, our study excluded patients with any chronic respiratory disease as they require long-term antibiotic treatment [54]. The EHR can only record the dates when prescriptions are made, but it cannot determine how closely patients adhere to their antibiotic regimen or the timing of repeated use. Similarly, there was no record of consultation type to indicate whether it was remote or face-to-face, so we could not determine whether the antibiotic prescription was evidence-based. Another constraint was that our study relied on AE categorised by hospital coding department [55]. In our analysis of AE, we only included diagnoses made upon admission, potentially leading to an underestimation of such events. This approach was taken to omit adverse events that might have occurred during a hospital stay due to medical interventions or treatments. An additional limitation was that our study only incorporated prescription data from primary care, excluding, for instance, hospitals or walk-in clinics. Nevertheless, as of 2021, 80.5% of antibiotic prescriptions in England are issued in primary care settings [3].

## Conclusions

In summary, our study provides a comprehensive analysis of the incidence of AEs following antibiotic prescriptions for common infections. We observed that the incidence rate of AEs fluctuated during the period from March 2020 to April 2021 and returned to pre-pandemic levels afterwards. Additionally, this study developed separate models for each type of infection, aiming to improve the accuracy in predicting calibration and discrimination. We found that most risk prediction models exhibited good calibration and discrimination levels. These findings highlight the necessity of cautious antibiotic prescribing and emphasise the need for further research to understand the complex factors influencing AE risk.

### Supplementary Information

Additional file 1: Tables S1- S5. Tab S1 . Codelists used for variable definition. Tab S2 . Patient characteristics for otitis externa, otitis media, and sinusitis patients. Summary statistics are presented as number (percentage) except where indicated. TabS3. Adverse event frequency by system following antibiotic treatment for common infections. TabS4. Coefficients for prediction models. TabS5. Model performance with calibration and discrimination (overall cohort). Figures S1-S6. Fig S1 . Kaplan–Meier plots for AE within 30 days after antibiotic treatment, showing cumulative survival probability of AE by period and infection type. Fig S2 . Period 1 cohort (pre-COVID): Adjusted hazard ratios for selected predictors, including health behavioral and clinical variables. Fig S3 . Overall cohort: Adjusted hazard ratios for selected predictors, including health behavioral and clinical variables. Fig S4 . Extended analysis for the overall cohort: Adjusted hazard ratios for selected predictors, including additional health behavioral and clinical variables. Fig S5 . Estimated log hazard ratios (HRs) against continuous age for different infections. FigS6. Calibration plot for Otitis externa/Otitis media/Sinusitis models. This plot displays observed survival probabilities (Y-axis) versus predicted survival probabilities (X-axis), generated from the validation cohort.

Additional file 2: Tripod Checklist.

## Data Availability

No datasets were generated or analysed during the current study.

## Abbreviations

ADR: Adverse drug reaction
AE: Adverse event
BMI: Body mass index
BNF: British National Formulary
CCI: Charlson Comorbidity Index
EHR: Electronic health record
HR: Hazard ratio
IMD: Index of Multiple Deprivation
LRTI: Lower respiratory tract infection
SGSS: Second Generation Surveillance System
SUS: Secondary Use Service
TRIPOD: Transparent Reporting of a Multivariable Prediction Model for Individual Prognosis or Diagnosis
URTI: Upper respiratory tract infection
UTI: Urinary tract infection
