# Supplementary material for: Risk of emergency hospital admission related to adverse events after antibiotic treatment in adults with a common infection: impact of COVID-19 and derivation and validation of risk prediction models
Source: BMC Med. 2024 Jul 2;22:277. doi: 10.1186/s12916-024-03480-2 (PMC11220965; doi:10.1186/s12916-024-03480-2)
Supplement: Supplementary file 1 — Additional file 1: Tables S1- S5. Tab S1 . Codelists used for variable definition. Tab S2 . Patient characteristics for otitis externa, otitis media, and sinusitis patients. Summary statistics are presented as number (percentage) except where indicated. TabS3. Adverse event frequency by system following antibiotic treatment for common infections. TabS4. Coefficients for prediction models. TabS5. Model performance with calibration and discrimination (overall cohort). Figures S1-S6. Fig S1 . Kaplan–Meier plots for AE within 30 days after antibiotic treatment, showing cumulative survival probability of AE by period and infection type. Fig S2 . Period 1 cohort (pre-COVID): Adjusted hazard ratios for selected predictors, including health behavioral and clinical variables. Fig S3 . Overall cohort: Adjusted hazard ratios for selected predictors, including health behavioral and clinical variables. Fig S4 . Extended analysis for the overall cohort: Adjusted hazard ratios for selected predictors, including additional health behavioral and clinical variables. Fig S5 . Estimated log hazard ratios (HRs) against continuous age for different infections. FigS6. Calibration plot for Otitis externa/Otitis media/Sinusitis models. This plot displays observed survival probabilities (Y-axis) versus predicted survival probabilities (X-axis), generated from the validation cohort. [file 12916_2024_3480_MOESM1_ESM.docx]

Supplement:

[Table 1. Code lists used for variable definition 2](#_Toc168857514)

[Table 2. Patient characteristics for otitis externa, otitis media and sinusitis patients. Summary statistics are number (percentage) except where indicated 3](#_Toc168857515)

[Table 3 Adverse event frequency by system following antibiotic treatment for common infections 4](#_Toc168857516)

[Table 4 Coefficients for prediction models 6](#_Toc168857517)

[Table 5. Model performance with calibration and discrimination (overall cohort) 10](#_Toc168857518)

[Figure 1. Kaplan-Meier plots for AE in 30 days after antibiotics. Plots show cumulative survival probability of AE by period and infection 11](#_Toc168858192)

[Figure 2. Period 1 cohort (pre-covid): Adjusted hazard ratios for selected predictors (including health behavioural and clinical variables) 13](#_Toc168858193)

[Figure 3. Overall cohort: Adjusted hazard ratios for selected predictors (including health behavioural and clinical variables) 15](#_Toc168858194)

[Figure 4. Overall cohort: Adjusted hazard ratios for selected predictors (including health behavioural and clinical variables 18](#_Toc168858195)

[Figure 5. Estimated log hazard ratios (HRs) against continuous age for different infection 20](#_Toc168858196)

[Figure 6 Calibration plot for Otitis externa/Otitis media/Sinusitis models. Calibration plot showing observed survival probabilities (Y-axis) versus predicted survival probabilities (X-axis). The plot was generated from the validation cohort. 22](#_Toc168858197)

**Table 1. Code lists used for variable definition**

1. Code lists are available at https://www.opencodelists.org/

2. Link: https://www.opencodelists.org/codelist/ add code list version

| **variable** | **Codelist version** |
| --- | --- |
| Ethnicity | https://codelists.opensafely.org/codelist/opensafely/ethnicity/2020-04-27/ |
| Adverse event | https://github.com/opensafely/amr-uom-brit/blob/ab_sideeffect_2/analysis/codelists.py |
| **Covid diagnosis** |  |
| Covid  (Primary care) | https://codelists.opensafely.org/codelist/opensafely/covid-identification-in-primary-care-probable-covid-clinical-code/24391856 |
| Covid positive test  (Primary care) | https://codelists.opensafely.org/codelist/opensafely/covid-identification-in-primary-care-probable-covid-positive-test/3d488b8b/ |
| Covid (primary care sequalae) | https://codelists.opensafely.org/codelist/opensafely/covid-identification-in-primary-care-probable-covid-sequelae/0b29a521/ |
| covid_codelist | codelist(["U071", "U072"], system="icd10") |
| confirmed_covid_codelist | codelist(["U071"], system="icd10") |
| suspected_covid_codelist | codelist(["U072"], system="icd10") |
| Smoking | "https://codelists.opensafely.org/codelist/opensafely/smoking-clear/2020-04-29/" |
| **Infection** |  |
| URTI | <https://codelists.opensafely.org/codelist/user/rriefu/cold_subset/0c46abca/>  <https://codelists.opensafely.org/codelist/user/rriefu/cough/6a0be66d/>  <https://codelists.opensafely.org/codelist/user/rriefu/throat/0880d98f/>  https://codelists.opensafely.org/codelist/user/rriefu/urti/6f716659/ |
| LRTI | https://codelists.opensafely.org/codelist/user/rriefu/lrti/5cda8555/ |
| Asthma diagnosis | "https://codelists.opensafely.org/codelist/opensafely/asthma-diagnosis/2020-04-15/"  https://codelists.opensafely.org/codelist/opensafely/asthma-oral-prednisolone-medication/2020-04-27/ |
| UTI | https://codelists.opensafely.org/codelist/user/rriefu/uti/5661f324/ |
| Chronic Respiratory Disease | https://www.opencodelists.org/codelist/opensafely/chronic-respiratory-disease/2020-04-10/ |
| Otitis externa | https://codelists.opensafely.org/codelist/user/rriefu/ot_externa/05ce1999/ |
| Otitis media | https://codelists.opensafely.org/codelist/user/rriefu/otmedia/3a9fbff9/ |
| Sinusitis | https://codelists.opensafely.org/codelist/user/rriefu/sinusits/21904cc4/ |
| **Comorbidities** | https://codelists.opensafely.org/codelist/opensafely/diabetes/2020-04-15/ |
| CCI | <https://codelists.opensafely.org/codelist/user/yayang/charlson01_cancer/796c49a5/>  <https://codelists.opensafely.org/codelist/user/yayang/charlson02_cvd/605cd670/>  https://codelists.opensafely.org/codelist/user/yayang/charlson03_copd/474d633b/  https://codelists.opensafely.org/codelist/user/yayang/charlson04_heart_failure/2e3df006/  https://codelists.opensafely.org/codelist/user/yayang/charlson05_connective_tissue/152e7cd1/  https://codelists.opensafely.org/codelist/user/yayang/charlson06_dementia/7c1f099b/  https://codelists.opensafely.org/codelist/user/yayang/charlson07_diabetes/630f9666/  https://codelists.opensafely.org/codelist/user/yayang/charlson08_diabetes_with_complications/4a002331/  https://codelists.opensafely.org/codelist/user/yayang/charlson09_hemiplegia/30f0affc/  https://codelists.opensafely.org/codelist/user/yayang/charlson10_hiv/17e13cc7/  https://codelists.opensafely.org/codelist/user/yayang/charlson11_metastatic_cancer/7ed1c991/  https://codelists.opensafely.org/codelist/user/yayang/charlson12_mild_liver/65c2565c/  https://codelists.opensafely.org/codelist/user/yayang/charlson13_mod_severe_liver/4cb2e327/  https://codelists.opensafely.org/codelist/user/yayang/charlson14_moderate_several_renaldiseae/33a36ff2/  https://codelists.opensafely.org/codelist/user/yayang/charlson15_mi/1a93fcbd/  https://codelists.opensafely.org/codelist/user/yayang/charlson16_peptic_ulcer/36562fe8/  https://codelists.opensafely.org/codelist/user/yayang/charlson17_peripheral_vascular/68751652/ |
| Antibacterial | https://codelists.opensafely.org/codelist/user/BillyZhongUOM/brit_new_dmd/792101bd |

**Table 2. Patient characteristics for otitis externa, otitis media and sinusitis patients. Summary statistics are number (percentage) except where indicated**

| Characteristic | Levels | Otitis externa | Otitis media | Sinusitis |
| --- | --- | --- | --- | --- |
| Total N |  | 339190 | 394825 | 627190 |
| EVENT | No | 338425 (99.8) | 394165 (99.8) | 625990 (99.8) |
|  | Yes | 765 (0.2) | 660 (0.2) | 1200 (0.2) |
| Age | Mean (SD) | 49.1 (18.0) | 45.8 (17.3) | 48.9 (15.8) |
|  | 18-39 | 118755 (35.0) | 166690 (42.2) | 202265 (32.2) |
|  | 40-49 | 58440 (17.2) | 69825 (17.7) | 125585 (20.0) |
|  | 50-59 | 62275 (18.4) | 67225 (17.0) | 129145 (20.6) |
|  | 60-69 | 46845 (13.8) | 47005 (11.9) | 97400 (15.5) |
|  | 70-79 | 34400 (10.1) | 30230 (7.7) | 56825 (9.1) |
|  | 80+ | 18475 (5.4) | 13850 (3.5) | 15970 (2.5) |
| Sex | Female | 220635 (65.0) | 260625 (66.0) | 464380 (74.0) |
|  | Male | 118555 (35.0) | 134200 (34.0) | 162810 (26.0) |
| IMD^1^ | 5 (least deprived) | 57510 (17.0) | 61810 (15.7) | 124615 (19.9) |
|  | 4 | 65425 (19.3) | 72590 (18.4) | 134430 (21.4) |
|  | 3 | 70950 (20.9) | 79320 (20.1) | 136015 (21.7) |
|  | 2 | 68970 (20.3) | 83600 (21.2) | 120620 (19.2) |
|  | 1 (most deprived) | 75810 (22.4) | 96845 (24.5) | 110545 (17.6) |
| Ethnicity^2^ | White | 295280 (87.1) | 335195 (84.9) | 553165 (88.2) |
|  | Mixed | 3065 (0.9) | 3950 (1.0) | 5970 (1.0) |
|  | South Asian | 22380 (6.6) | 31500 (8.0) | 34605 (5.5) |
|  | Black | 3945 (1.2) | 5420 (1.4) | 7320 (1.2) |
|  | Other | 4425 (1.3) | 5700 (1.4) | 7490 (1.2) |
|  | Unknown | 10090 (3.0) | 13060 (3.3) | 18635 (3.0) |
| Region | East of England | 85295 (25.1) | 105025 (26.6) | 170635 (27.2) |
|  | North East | 16525 (4.9) | 18465 (4.7) | 25435 (4.1) |
|  | North West | 34185 (10.1) | 41600 (10.5) | 65040 (10.4) |
|  | Yorkshire and the Humber | 51645 (15.2) | 65720 (16.6) | 95510 (15.2) |
|  | East Midlands | 66485 (19.6) | 75355 (19.1) | 115345 (18.4) |
|  | West Midlands | 13015 (3.8) | 17910 (4.5) | 22235 (3.5) |
|  | London | 14370 (4.2) | 16180 (4.1) | 25140 (4.0) |
|  | South East | 21575 (6.4) | 20560 (5.2) | 40170 (6.4) |
|  | South West | 35905 (10.6) | 33830 (8.6) | 67315 (10.7) |
|  | Unknown | 195 (0.1) | 180 (0.0) | 370 (0.1) |
| Smoking^3^ | Never and unknown | 144845 (42.7) | 178380 (45.2) | 279530 (44.6) |
|  | Former | 127465 (37.6) | 137270 (34.8) | 244635 (39.0) |
|  | Current | 66880 (19.7) | 79180 (20.1) | 103025 (16.4) |
| BMI^4^ | Not obese | 217165 (64.0) | 259410 (65.7) | 432430 (68.9) |
|  | Obese I (30-34.9 kg/m2) | 62110 (18.3) | 68805 (17.4) | 107415 (17.1) |
|  | Obese II (35-39.9 kg/m2) | 32650 (9.6) | 36550 (9.3) | 50890 (8.1) |
|  | Obese III (40+ kg/m2) | 27265 (8.0) | 30065 (7.6) | 36455 (5.8) |
| CCI^5^ | No | 235000 (69.3) | 288285 (73.0) | 445030 (71.0) |
|  | low | 96340 (28.4) | 100315 (25.4) | 173920 (27.7) |
|  | medium | 7335 (2.2) | 5875 (1.5) | 7900 (1.3) |
|  | high | 500 (0.1) | 340 (0.1) | 335 (0.1) |
|  | very high | 20 (0.0) | 15 (0.0) | <10 (0.0) |
| Antibiotic history (3 years)^6^ | 0 | 86875 (25.6) | 114015 (28.9) | 139600 (22.3) |
|  | 1 | 57110 (16.8) | 71895 (18.2) | 103935 (16.6) |
|  | 2-3 | 73760 (21.7) | 88120 (22.3) | 142205 (22.7) |
|  | 4+ | 121445 (35.8) | 120800 (30.6) | 241450 (38.5) |
| Antibiotic use (30 days)^7^ | No | 248705 (73.3) | 293115 (74.2) | 480800 (76.7) |
|  | Yes | 90490 (26.7) | 101715 (25.8) | 146390 (23.3) |

To reduce the risk of secondary disclosure, all counted numbers in the baseline table were rounded to the nearest five.

1. IMD (Index of Multiple Deprivation) quintile measured from patient-level address

2. Ethnicity in line with 2001 Census categories

3. Smoking status identified from the most recent clinical records

4. BMI, Body Mass Index groups based on the NICE definitions

5. The Charlson Comorbidities Index (CCI) is a method of categorizing comorbidities of patients based on the International Classification of Diseases (ICD) diagnosis codes found in administrative data. It includes 17 weighted conditions such as Myocardial infarction, Congestive heart failure, Peripheral vascular disease, Cerebrovascular disease, Dementia, Chronic pulmonary disease, Connective tissue disease, Ulcer disease, Mild liver disease, Diabetes, Hemiplegia, Moderate or severe renal disease, Diabetes with complications, Any malignancy (including leukaemia and lymphoma), Moderate or severe liver disease, Metastatic solid tumour, and AIDS.

6. The patient's antibiotic prescription history spans from three years plus 90 days, up until 90 days prior to the outcome date.

7. The binary variable indicating if there were any antibiotic treatments administered in the 30 days preceding the index date.

**Table 3 Adverse event frequency by system following antibiotic treatment for common infections**

|  | UTI | | URTI | | LRTI | |
| --- | --- | --- | --- | --- | --- | --- |
|  | N | % | N | % | N | % |
| Hematologic | 245 | 1.2 | 137 | 1.9 | 146 | 1.4 |
| Circulatory system | 3256 | 16.4 | **1634** | **22.7** | **2896** | **27.5** |
| Digestive system | 2195 | 11.0 | 764 | 10.6 | 1070 | 10.2 |
| Endocrine | 2820 | 14.2 | 630 | 8.8 | 1149 | 10.9 |
| Eye and ear | 76 | 0.4 | 42 | 0.6 | 40 | 0.4 |
| Genitourinary system (other than kidney) | 2280 | 11.5 | 193 | 2.7 | 192 | 1.8 |
| Liver | 135 | 0.7 | 30 | 0.4 | 54 | 0.5 |
| Nervous system | 38 | 0.2 | 18 | 0.3 | 25 | 0.2 |
| Musculoskeletal system and connective tissue | 88 | 0.4 | 32 | 0.4 | 55 | 0.5 |
| Poisoning | 171 | 0.9 | 113 | 1.6 | 103 | 1.0 |
| Renal | **4242** | **21.3** | 599 | 8.3 | 1291 | 12.3 |
| Respiratory system | 864 | 4.3 | 1529 | 21.3 | 2101 | 19.9 |
| Skin and subcutaneous tissue | 402 | 2.0 | 229 | 3.2 | 165 | 1.6 |
| Others | 3101 | 15.6 | 1238 | 17.2 | 1247 | 11.8 |

**Table 4 Coefficients for prediction models**

| Infection | UTI | LRTI | URTI | sinusitis | ot media | ot_externa |
| --- | --- | --- | --- | --- | --- | --- |
| Baseline Hazard in Day 30 | 0.9947264 | 0.9947244 | 0.9971367 | 0.9980877 | 0.9985813 | 0.9988678 |
| sexMale | 0.559907616 | 0.124866692 | 0.130721921 | 0.032680274 | -0.065847369 | 0.113289141 |
| age3_splinetraining | 0.012358007 | 0.01043078 | 0.008953899 | -0.0267161 | 0.001354512 | -0.026703338 |
| age3_splinetraining | 0.022008179 | 0.017395488 | 0.019839513 | 0.046125948 | 0.028741555 | 0.060480032 |
| regionNorth East | 0.200836267 | -0.065264835 | 0.072283188 | -0.056340988 | 0.096084286 | -0.095238668 |
| regionNorth West | -0.169406445 | 0.027213983 | 0.034400876 | -0.077876548 | -0.256967555 | 0.041300882 |
| regionYorkshire and the Humber | -0.09322467 | -0.106009823 | -0.148237427 | -0.075440509 | -0.642281696 | 0.001013196 |
| regionEast Midlands | -0.080728318 | -0.004698311 | 0.071784055 | 0.181734261 | 0.121952989 | 0.148913997 |
| regionWest Midlands | 0.062995865 | 0.058435985 | 0.08624801 | 0.238803824 | 0.152046329 | 0.084690189 |
| regionLondon | 0.015234279 | 0.017871507 | 0.005576025 | -0.111726291 | 0.201140265 | -0.233284558 |
| regionSouth East | -0.017932029 | -0.013909115 | -0.013193099 | 0.15186796 | 0.383620652 | -0.150022437 |
| regionSouth West | -0.077393468 | 0.063687681 | -0.140523175 | 0.081812083 | 0.020059939 | 0.33412592 |
| region Unknown | 0.01879864 | 0.161113588 | -0.079260447 | -13.29245816 | -11.60751297 | -16.14149436 |
| imd4 | -0.014971687 | -0.015343854 | 0.075089855 | 0.17485827 | -0.153889895 | -0.477772175 |
| imd3 | 0.071413161 | -0.072416412 | 0.076001301 | -0.066750902 | 0.079498195 | 0.014984132 |
| imd2 | 0.136490333 | -0.022211527 | 0.055612999 | 0.121243382 | -0.01042777 | 0.258086297 |
| imd1 (most deprived) | 0.191851788 | 0.051229188 | 0.179361565 | 0.320374523 | 0.24532822 | 0.038508505 |
| imdUnknown | 0.173288877 | 0.131677323 | 0.10628761 | 0.620345653 | 1.228610336 | -15.42842453 |
| ethnicityMixed | -0.160640972 | 0.170596155 | -0.159657679 | 0.474130132 | 0.436634237 | -15.59401282 |
| ethnicitySouth Asian | -0.100231533 | -0.037360724 | -0.020088558 | 0.270252712 | 0.208907251 | 0.071531094 |
| ethnicityBlack | 0.351293154 | -0.198040086 | -0.075453707 | 0.250356275 | 1.388540302 | 0.821126877 |
| ethnicityOther | -0.218735573 | -0.178997063 | -0.440493436 | 0.658083565 | -0.826642938 | -0.744905077 |
| ethnicityUnknown | -0.910034372 | -1.009782144 | -1.486595415 | -1.227656305 | -1.53698456 | -16.03135347 |
| bmiObese I (30-34.9 kg/m2) | -0.028079712 | 0.038695246 | -0.038324169 | -0.040011423 | 0.121528946 | 0.111897842 |
| bmiObese II (35-39.9 kg/m2) | 0.097739213 | 0.074009275 | -0.004031511 | 0.199420188 | -0.348566713 | -0.19397044 |
| bmiObese III (40+ kg/m2) | 0.369610548 | 0.357155923 | 0.426180798 | -0.126837694 | -0.258269879 | 0.410606561 |
| smoking_status_combFormer | 0.007227612 | 0.018343963 | 0.040299586 | 0.099646817 | 0.208046387 | -0.03339673 |
| smoking_status_combCurrent | 0.215445258 | 0.076071961 | 0.137220766 | 0.337218188 | 0.207922027 | -0.097962662 |
| charlsonGrphigh | 1.413565704 | 1.180945013 | 1.120691297 | -13.62444964 | 1.416375421 | 1.008636961 |
| charlsonGrplow | 0.508411907 | 0.371066214 | 0.428245365 | 0.277527004 | 0.47355996 | 0.436319905 |
| charlsonGromedium | 0.916204033 | 0.796322346 | 0.898874391 | 0.745061535 | 0.69794502 | 0.691839056 |
| charlsonGrovery high | 1.667353522 | 1.07632762 | 1.403506446 | -13.74646203 | -12.40215295 | -17.95372229 |
| ab_30dTRUE | 0.173451722 | 0.354267843 | 0.52732736 | 0.455265671 | 0.134615095 | 0.274406139 |
| ab_3yr1 | -0.056584673 | 4.92E-04 | 0.14751894 | 0.294665568 | 0.500971901 | 0.567714887 |
| ab_3yr2-3 | -0.012414542 | -0.027502275 | 0.020398654 | 0.228532924 | 0.571371943 | 0.699071265 |
| ab_3yr4+ | -0.045518942 | 0.118373901 | 0.105457011 | 0.428782878 | 0.787618921 | 0.983584091 |

| **Infection** | **UTI** | **LRTI** | **URTI** | **sinusitis** | **otmedia** | **ot_externa** |
| --- | --- | --- | --- | --- | --- | --- |
| **sexMale** | 0.559907616 | 0.124866692 | 0.130721921 | 0.032680274 | -0.065847369 | 0.113289141 |
| **age3_splinetraining** | 0.012358007 | 0.01043078 | 0.008953899 | -0.0267161 | 1.31E-04 | -0.026703338 |
| **age3_splinetraining** | 0.022008179 | 0.017395488 | 0.019839513 | 0.046125948 | 0.028741555 | 0.060480032 |
| **regionNorth East** | 0.200836267 | -0.065264835 | 0.072283188 | -0.056340988 | 0.096084286 | -0.095238668 |
| **regionNorth West** | -0.169406445 | 0.027213983 | 0.034400876 | -0.077876548 | -0.256967555 | 0.041300882 |
| **regionYorkshire and the Humber** | -0.09322467 | -0.106009823 | -0.148237427 | -0.075440509 | -0.642281696 | 0.001013196 |
| **regionEast Midlands** | -0.080728318 | -0.004698311 | 0.071784055 | 0.181734261 | 0.121952989 | 0.148913997 |
| **regionWest Midlands** | 0.062995865 | 0.058435985 | 0.08624801 | 0.238803824 | 0.152046329 | 0.084690189 |
| **regionLondon** | 0.015234279 | 0.017871507 | 0.005576025 | -0.111726291 | 0.201140265 | -0.233284558 |
| **regionSouth East** | -0.017932029 | -0.013909115 | -0.013193099 | 0.15186796 | 0.383620652 | -0.150022437 |
| **regionSouth West** | -0.077393468 | 0.063687681 | -0.140523175 | 0.081812083 | 0.020059939 | 0.33412592 |
| **region Unknown** | 0.01879864 | 0.161113588 | -0.079260447 | -13.29245816 | -11.60751297 | -16.14149436 |
| **imd4** | -0.014971687 | -0.015343854 | 0.075089855 | 0.17485827 | -0.153889895 | -0.477772175 |
| **imd3** | 0.071413161 | -0.072416412 | 0.076001301 | -0.066750902 | 0.079498195 | 0.014984132 |
| **imd2** | 0.136490333 | -0.022211527 | 0.055612999 | 0.121243382 | -0.01042777 | 0.258086297 |
| **imd1 (most deprived)** | 0.191851788 | 0.051229188 | 0.179361565 | 0.320374523 | 0.24532822 | 0.038508505 |
| **imdUnknown** | 0.173288877 | 0.131677323 | 0.10628761 | 0.620345653 | 1.228610336 | -15.42842453 |
| **ethnicityMixed** | -0.160640972 | 0.170596155 | -0.159657679 | 0.474130132 | 0.436634237 | -15.59401282 |
| **ethnicitySouth Asian** | -0.100231533 | -0.037360724 | -0.020088558 | 0.270252712 | 0.208907251 | 0.071531094 |
| **ethnicityBlack** | 0.351293154 | -0.198040086 | -0.075453707 | 0.250356275 | 1.388540302 | 0.821126877 |
| **ethnicityOther** | -0.218735573 | -0.178997063 | -0.440493436 | 0.658083565 | -0.826642938 | -0.744905077 |
| **ethnicityUnknown** | -0.910034372 | -1.009782144 | -1.486595415 | -1.227656305 | -1.53698456 | -16.03135347 |
| **bmiUnderweight (<18.5 kg/m2)** | NA | NA | NA | *NA* | NA | NA |
| **bmiOverweight (25-29.9 kg/m2)** | NA | NA | NA | NA | NA | NA |
| **bmiObese I (30-34.9 kg/m2)** | -0.028079712 | 0.038695246 | -0.038324169 | -0.040011423 | 0.121528946 | 0.111897842 |
| **bmiObese II (35-39.9 kg/m2)** | 0.097739213 | 0.074009275 | -0.004031511 | 0.199420188 | -0.348566713 | -0.19397044 |
| **bmiObese III (40+ kg/m2)** | 0.369610548 | 0.357155923 | 0.426180798 | -0.126837694 | -0.258269879 | 0.410606561 |
| **smoking_status_combFormer** | 0.007227612 | 0.018343963 | 0.040299586 | 0.099646817 | 0.208046387 | -0.03339673 |
| **smoking_status_combCurrent** | 0.215445258 | 0.076071961 | 0.137220766 | 0.337218188 | 0.207922027 | -0.097962662 |
| **charlsonGrphigh** | 1.413565704 | 1.180945013 | 1.120691297 | -13.62444964 | 1.416375421 | 1.008636961 |
| **charlsonGrplow** | 0.508411907 | 0.371066214 | 0.428245365 | 0.277527004 | 0.47355996 | 0.436319905 |
| **charlsonGromedium** | 0.916204033 | 0.796322346 | 0.898874391 | 0.745061535 | 0.69794502 | 0.691839056 |
| **charlsonGrovery high** | 1.667353522 | 1.07632762 | 1.403506446 | -13.74646203 | -12.40215295 | -17.95372229 |
| **ab_30dTRUE** | 0.173451722 | 0.354267843 | 0.52732736 | 0.455265671 | 0.134615095 | 0.274406139 |
| **ab_3yr1** | -0.056584673 | 4.92E-04 | 0.14751894 | 0.294665568 | 0.500971901 | 0.567714887 |

**Table 5. Model performance with calibration and discrimination (overall cohort)**

| Infection |  | C-statistic | Calibration slope |
| --- | --- | --- | --- |
| UTI | Development | 0.76 (0.76-0.77) | 0.999 |
|  | Validation | 0.76 (0.75-0.77) | 1.011 |
| URTI | Development | 0.73 (0.72-0.74) | 1.000 |
|  | Validation | 0.73 (0.72-0.75) | 1.022 |
| LRTI | Development | 0.70 (0.69-0.71) | 1.000 |
|  | Validation | 0.70 (0.68-0.71) | 0.983 |
| Otitis externa | Development | 0.75 (0.72-0.78) | 1.000 |
|  | Validation | 0.72 (0.66-0.78) | 0.920 |
| Otitis media | Development | 0.72 (0.68-0.76) | 1.000 |
|  | Validation | 0.70 (0.62-0.77) | 0.864 |
| Sinusitis | Development | 0.65 (0.62-0.67) | 1.000 |
|  | Validation | 0.69 (0.65-0.74) | 1.169 |

a. Otitis externa patients b. Otitis media patients


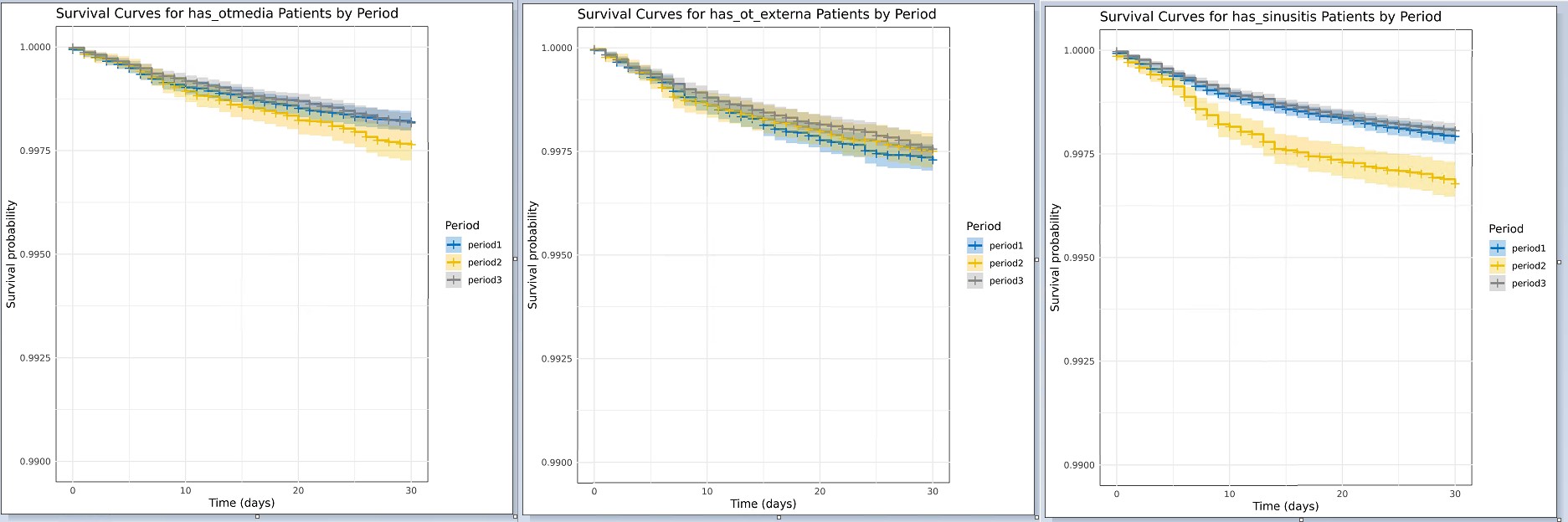

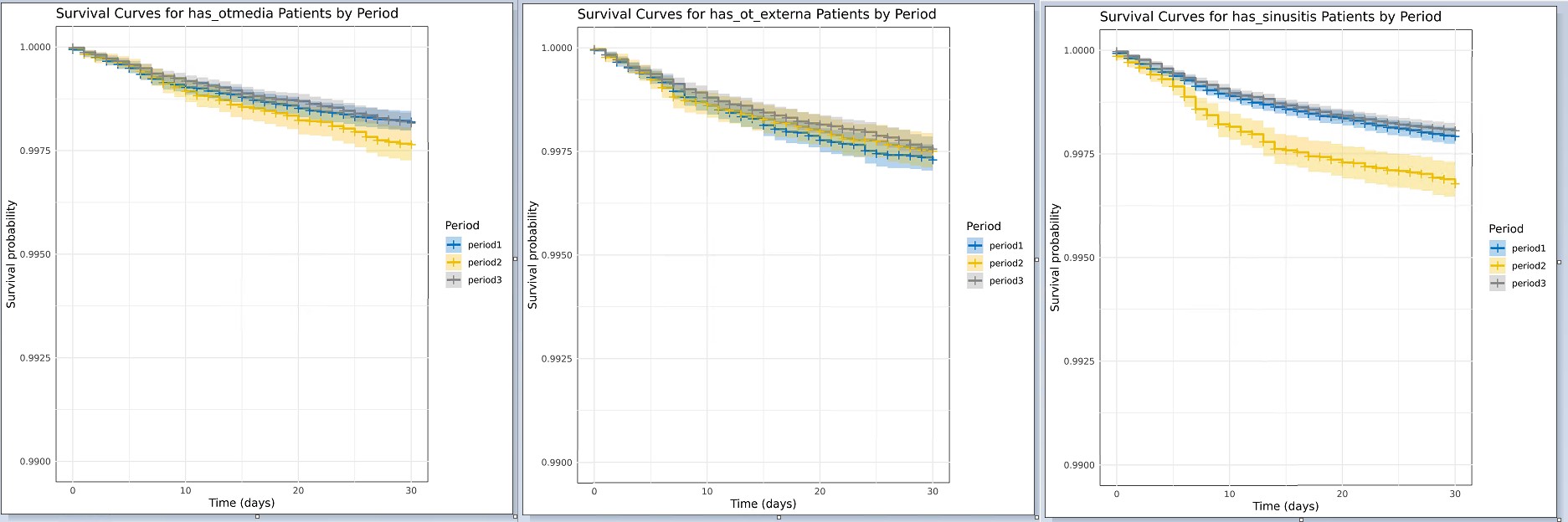


c. Sinusitis patients


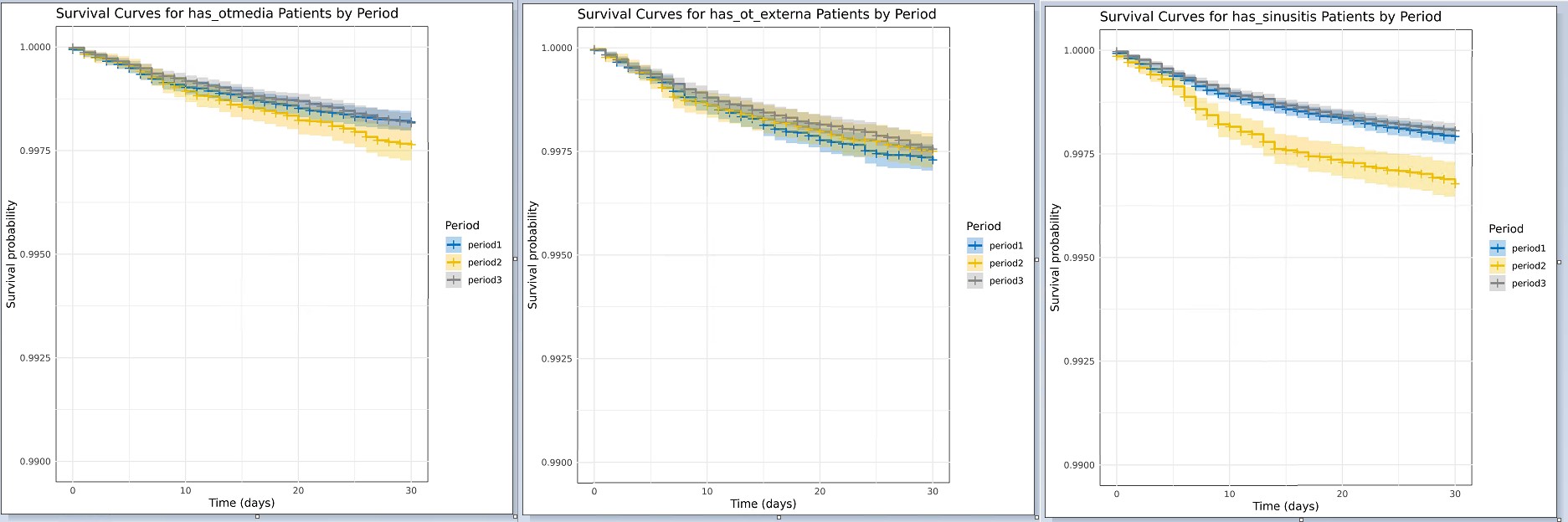


**Figure 1. Kaplan-Meier plots for AE in 30 days after antibiotics. Plots show cumulative survival probability of AE by period and infection**


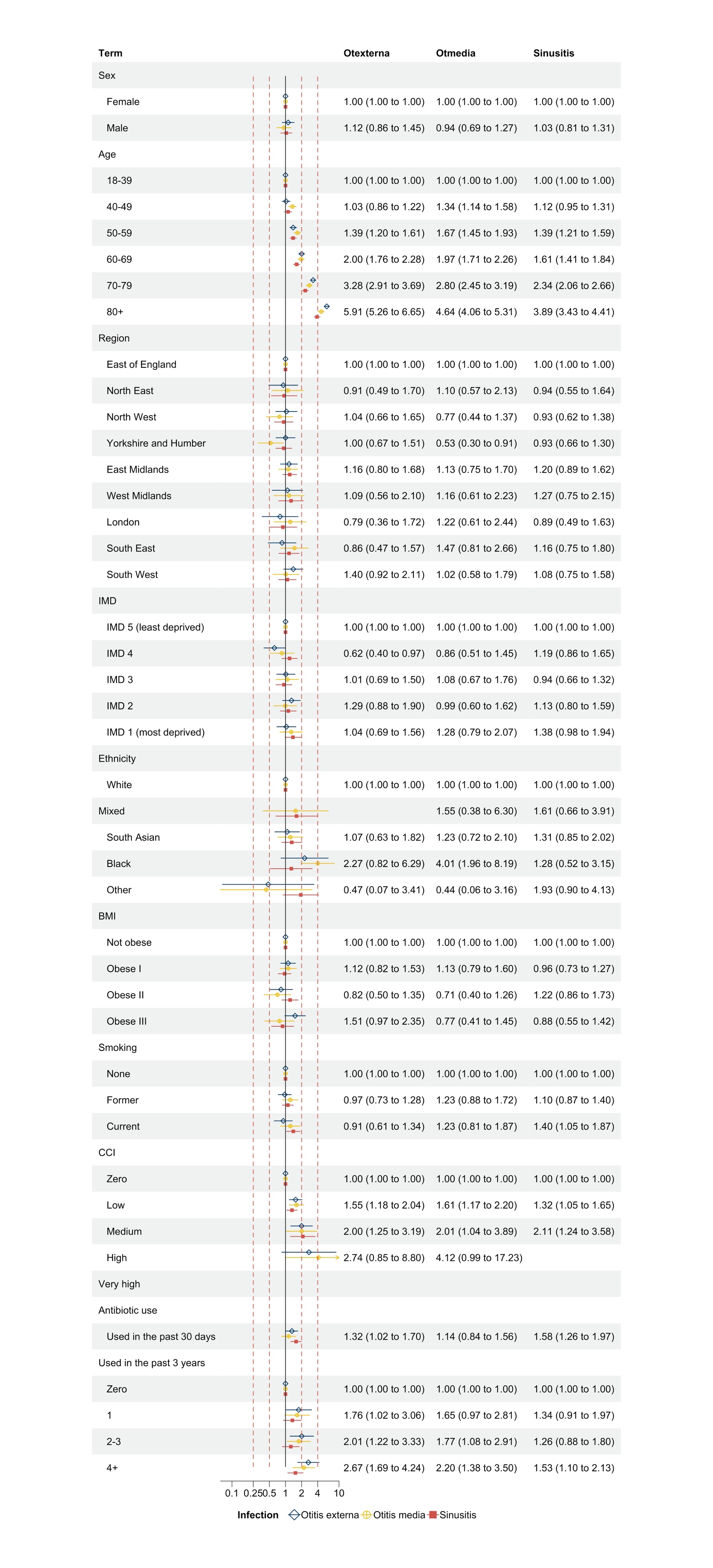


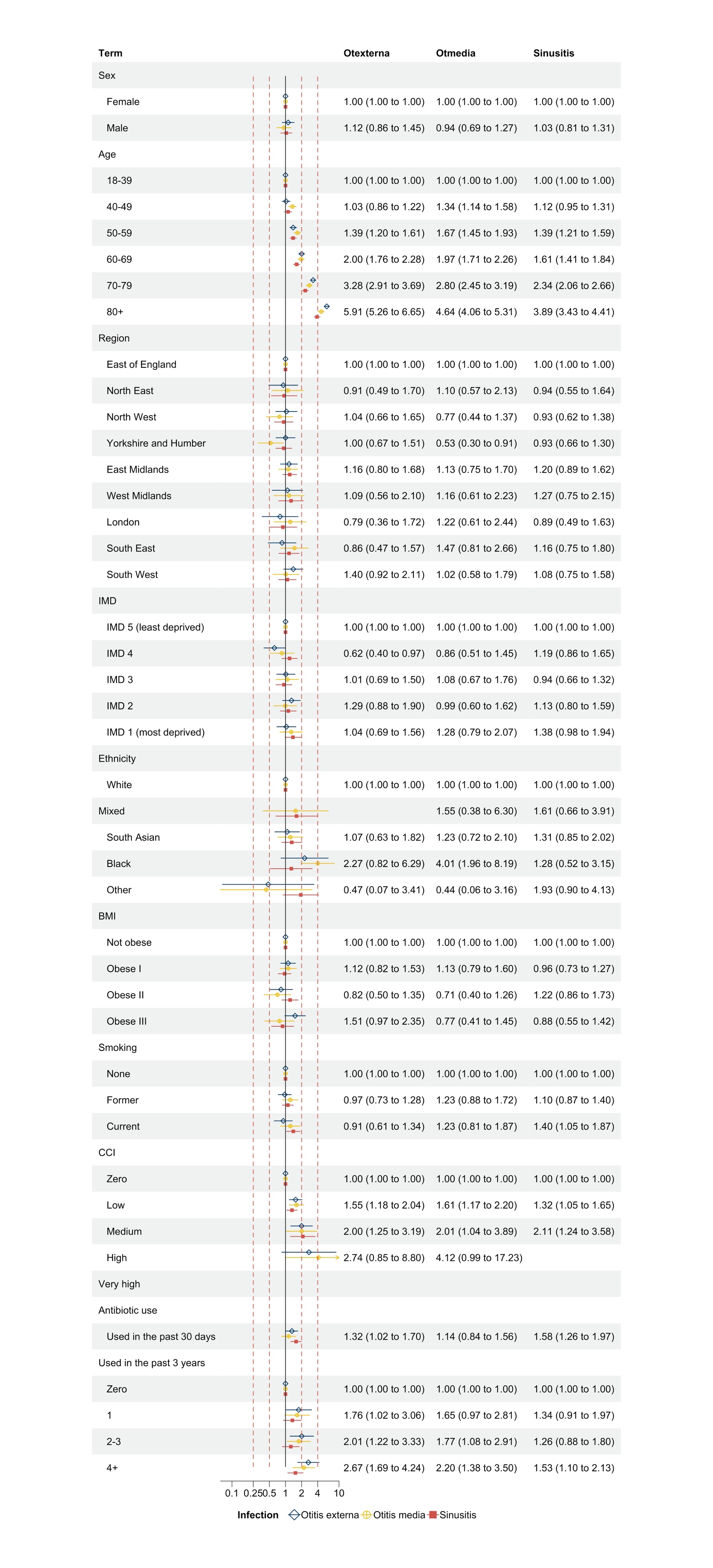


**Figure 2. Period 1 cohort (pre-covid): Adjusted hazard ratios for selected predictors (including health behavioural and clinical variables)**

The Index of Multiple Deprivation (IMD) quintile was derived from the patient's residential address.

Body Mass Index (BMI) refers to a calculation of body fat based on height and weight. obese I (30-34.9 kg/m2), obese II (35-39.9 kg/m2), and obese III (≥40kg/m2)

The Charlson Comorbidities Index (CCI) is a method of categorizing comorbidities of patients based on the International Classification of Diseases (ICD) diagnosis codes found in administrative data. It includes 17 weighted conditions such as Myocardial infarction, Congestive heart failure, Peripheral vascular disease, Cerebrovascular disease, Dementia, Chronic pulmonary disease, Connective tissue disease, Ulcer disease, Mild liver disease, Diabetes, Hemiplegia, Moderate or severe renal disease, Diabetes with complications, Any malignancy (including leukaemia and lymphoma), Moderate or severe liver disease, Metastatic solid tumour, and AIDS.

Used in the past 30 days: The binary variable indicating if there was any antibiotic treatments administered in the 30 days preceding the index date.

Used in the past 3 years: The patient's antibiotic prescription history spans from three years plus 90 days, up until 90 days prior to the outcome date.

Reference groups: Sex: Female, Age: 18-39, Region: East of England, IMD quintile: the least deprived quintile (IMD 5), Ethnicity: white ,BMI: Not obese (<30 kg/m2) Smoking: None (Smoking status identified from the most recent clinical records), CCI: Zero, Antibiotic use: used in the past 30 days: No, used in the past 3 years: zero.


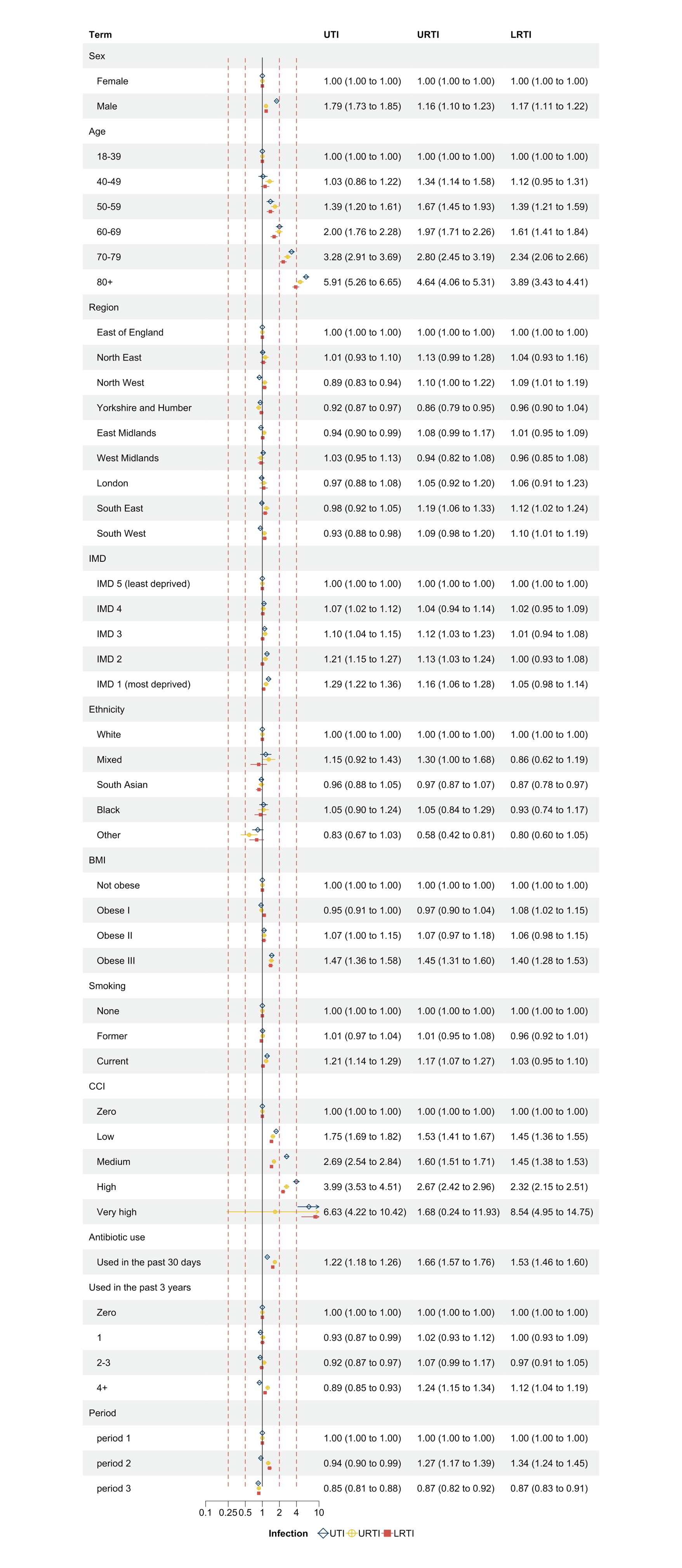


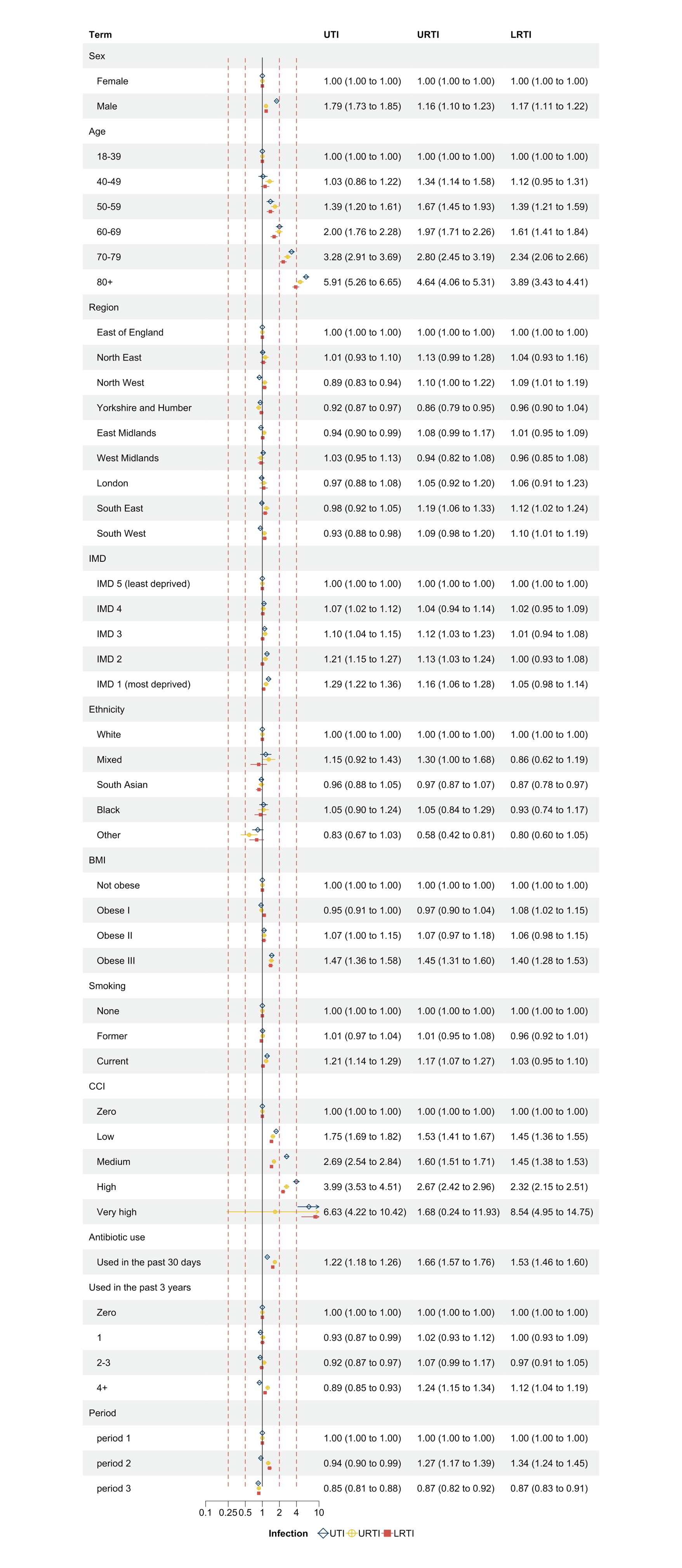


**Figure 3. Overall cohort: Adjusted hazard ratios for selected predictors (including health behavioural and clinical variables)**

The Index of Multiple Deprivation (IMD) quintile was derived from the patient's residential address.

Body Mass Index (BMI) refers to a calculation of body fat based on height and weight. obese I (30-34.9 kg/m2), obese II (35-39.9 kg/m2), and obese III (≥40kg/m2)

The Charlson Comorbidities Index (CCI) is a method of categorizing comorbidities of patients based on the International Classification of Diseases (ICD) diagnosis codes found in administrative data. It includes 17 weighted conditions such as Myocardial infarction, Congestive heart failure, Peripheral vascular disease, Cerebrovascular disease, Dementia, Chronic pulmonary disease, Connective tissue disease, Ulcer disease, Mild liver disease, Diabetes, Hemiplegia, Moderate or severe renal disease, Diabetes with complications, Any malignancy (including leukaemia and lymphoma), Moderate or severe liver disease, Metastatic solid tumour, and AIDS.

Used in the past 30 days: The binary variable indicating if there was any antibiotic treatments administered in the 30 days preceding the index date.

Used in the past 3 years: The patient's antibiotic prescription history spans from three years plus 90 days, up until 90 days prior to the outcome date.

Period 1: before COVID-19: 2019-01-01 to 2020-03-25 Period 2: implementation of national lockdown: 2020-03-26 to 2021-03-08

Period 3: after national lockdown: 2021-03-09 to 2022-06-30.

Reference groups: Sex: Female, Age: 18-39, Region: East of England, IMD quintile: the least deprived quintile (IMD 5), Ethnicity: white ,BMI: Not obese (<30 kg/m2) Smoking: None (Smoking status identified from the most recent clinical records), CCI: Zero, Antibiotic use: used in the past 30 days: No, used in the past 3 years: zero. Period: Period 1


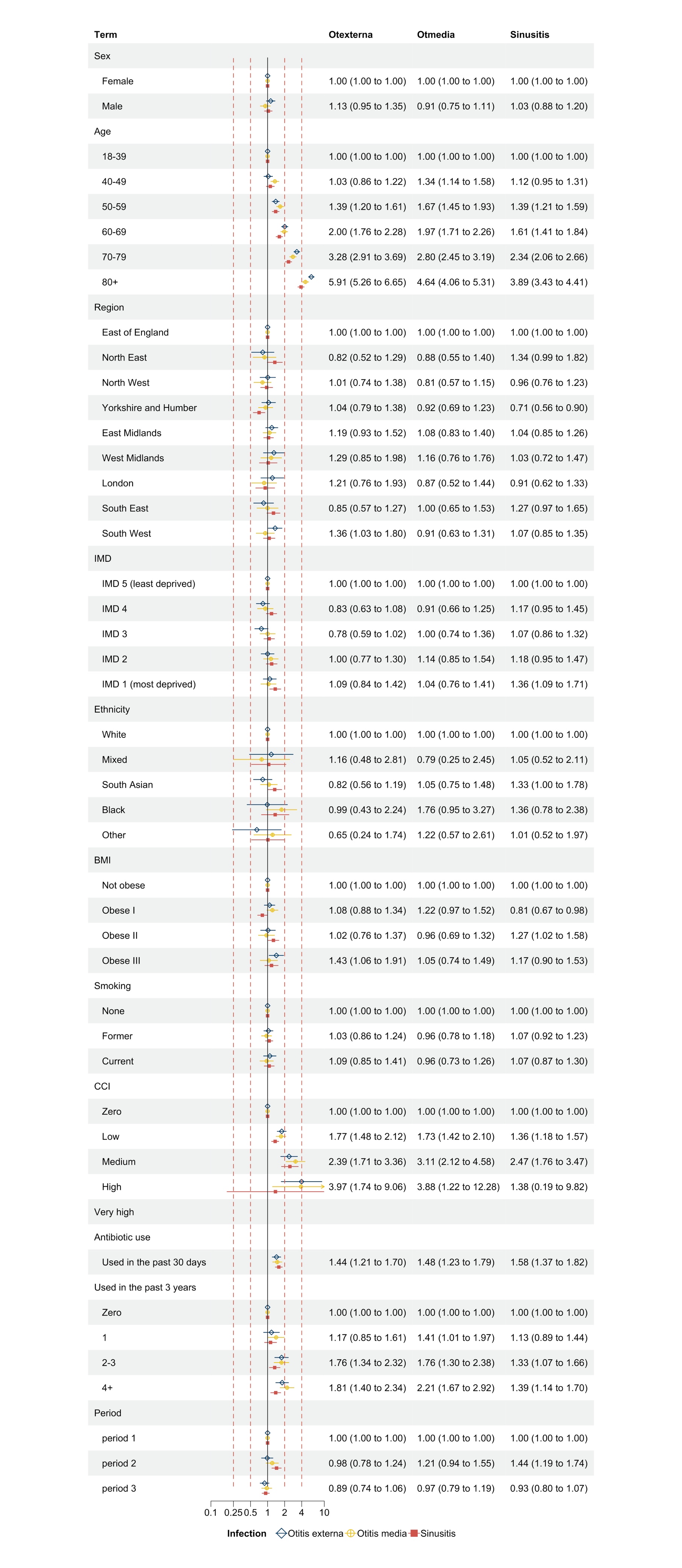


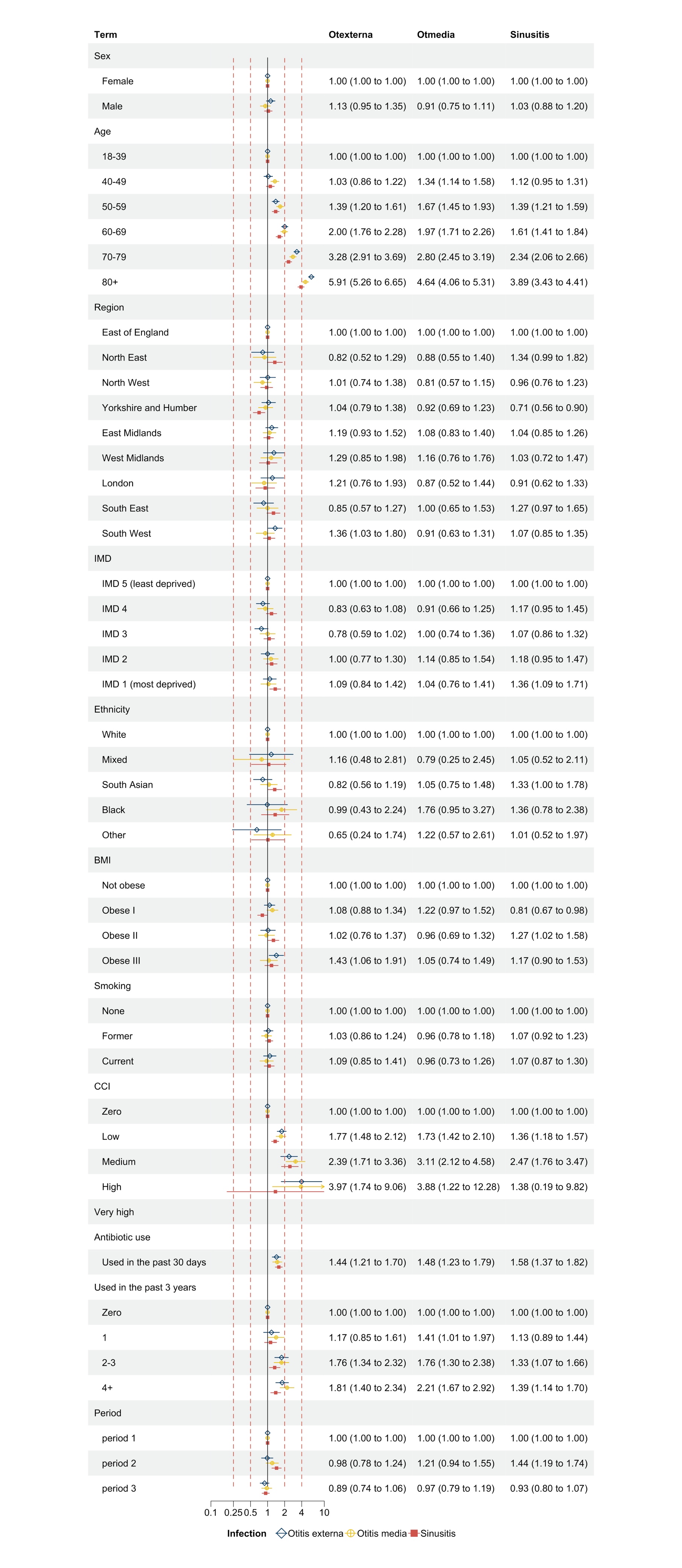


**Figure 4. Overall cohort: Adjusted hazard ratios for selected predictors (including health behavioural and clinical variables**

The Index of Multiple Deprivation (IMD) quintile was derived from the patient's residential address.

Body Mass Index (BMI) refers to a calculation of body fat based on height and weight. obese I (30-34.9 kg/m2), obese II (35-39.9 kg/m2), and obese III (≥40kg/m2)

The Charlson Comorbidities Index (CCI) is a method of categorizing comorbidities of patients based on the International Classification of Diseases (ICD) diagnosis codes found in administrative data. It includes 17 weighted conditions such as Myocardial infarction, Congestive heart failure, Peripheral vascular disease, Cerebrovascular disease, Dementia, Chronic pulmonary disease, Connective tissue disease, Ulcer disease, Mild liver disease, Diabetes, Hemiplegia, Moderate or severe renal disease, Diabetes with complications, Any malignancy (including leukaemia and lymphoma), Moderate or severe liver disease, Metastatic solid tumour, and AIDS.

Used in the past 30 days: The binary variable indicating if there was any antibiotic treatments administered in the 30 days preceding the index date.

Used in the past 3 years: The patient's antibiotic prescription history spans from three years plus 90 days, up until 90 days prior to the outcome date.

Period 1: before COVID-19: 2019-01-01 to 2020-03-25 Period 2: implementation of national lockdown: 2020-03-26 to 2021-03-08

Period 3: after national lockdown: 2021-03-09 to 2022-06-30.

Reference groups: Sex: Female, Age: 18-39, Region: East of England, IMD quintile: the least deprived quintile (IMD 5), Ethnicity: white ,BMI: Not obese (<30 kg/m2) Smoking: None (Smoking status identified from the most recent clinical records), CCI: Zero, Antibiotic use: used in the past 30 days: No, used in the past 3 years: zero. Period: Period 1


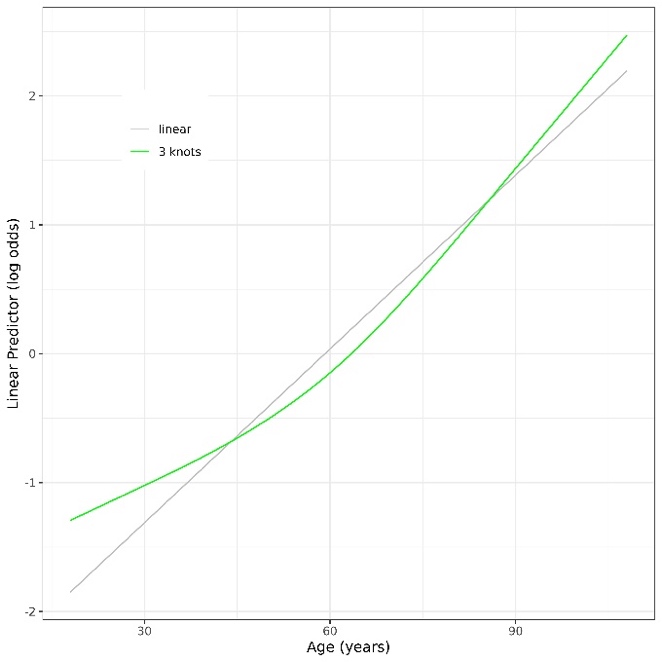

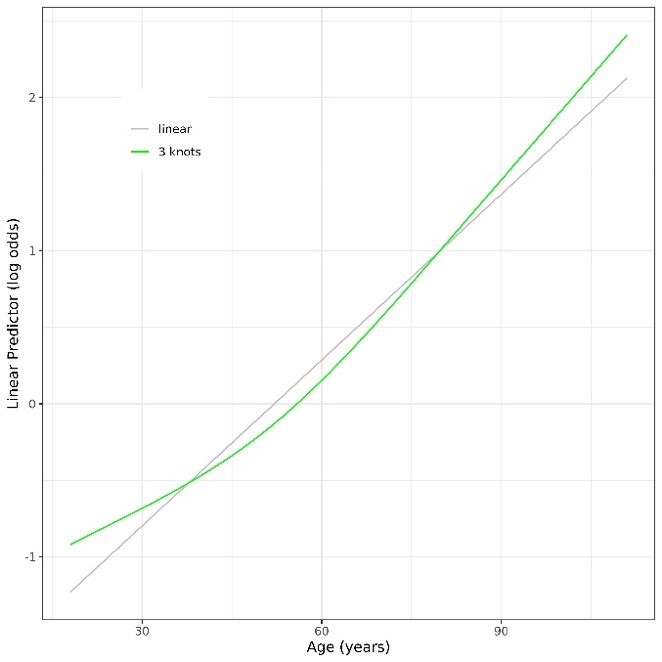


a.UTI b.URTI


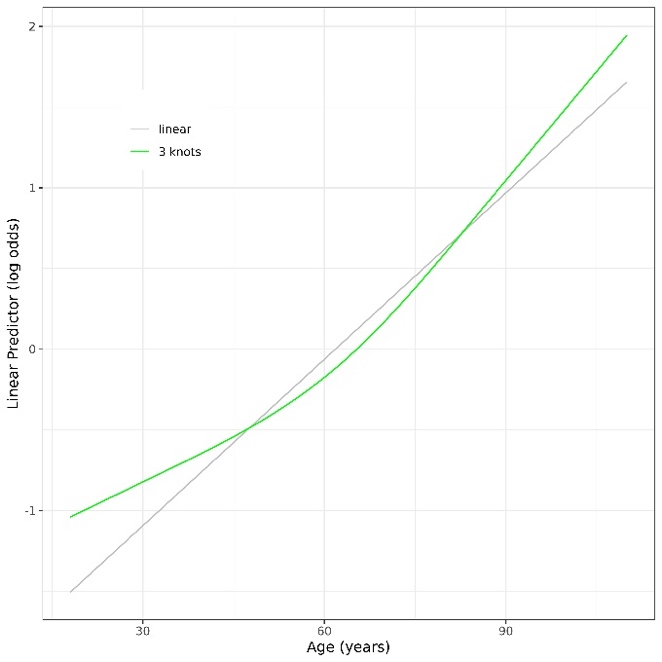

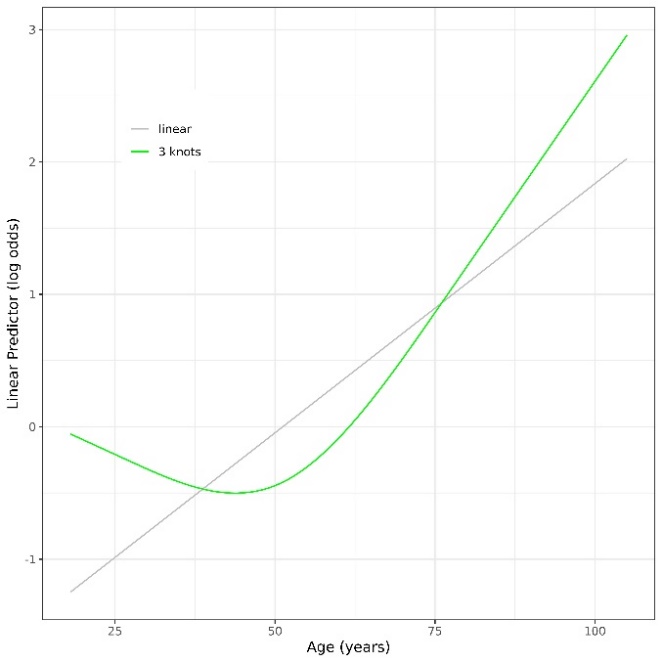


c.LRTI d.Otitis externa


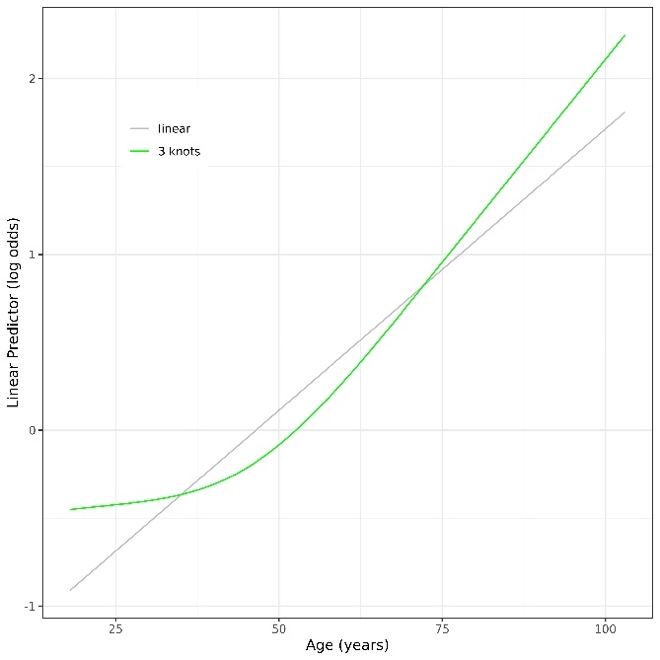

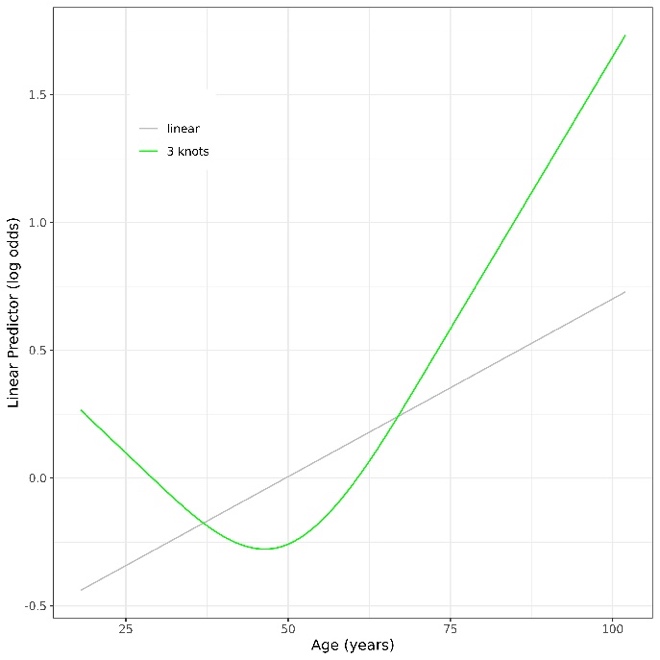


e. Otitis media f. Sinusitis

**Figure 5. Estimated log hazard ratios (HRs) against continuous age for different infection**


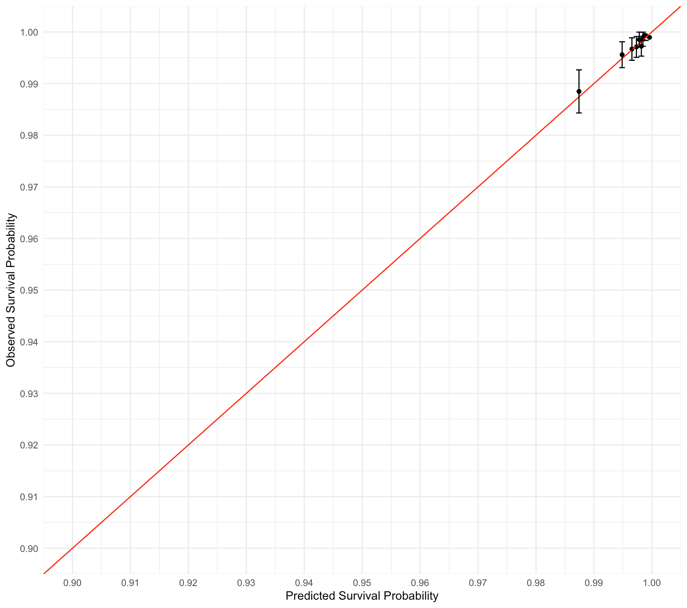

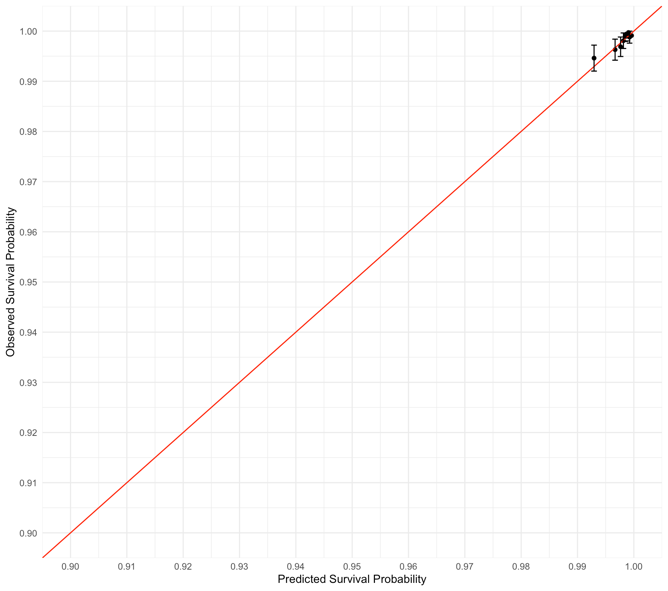


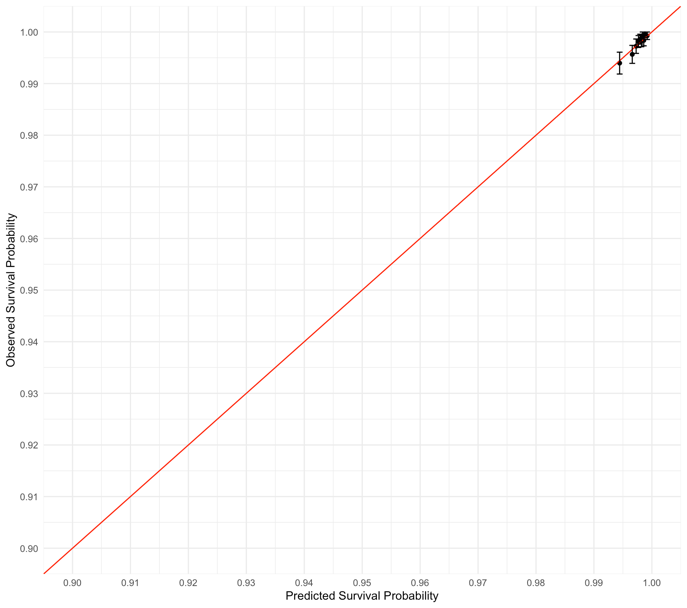


**Figure 6 Calibration plot for Otitis externa/Otitis media/Sinusitis models. Calibration plot showing observed survival probabilities (Y-axis) versus predicted survival probabilities (X-axis). The plot was generated from the validation cohort.**
